# Carbon Based Nanodots in Early Diagnosis of Cancer

**DOI:** 10.3389/fchem.2021.669169

**Published:** 2021-05-24

**Authors:** Gurpal Singh, Harinder Kaur, Akanksha Sharma, Joga Singh, Hema Kumari Alajangi, Santosh Kumar, Neha Singla, Indu Pal Kaur, Ravi Pratap Barnwal

**Affiliations:** ^1^ University Institute of Pharmaceutical Sciences, Panjab University, Chandigarh, India; ^2^ Department of Biophysics, Panjab University, Chandigarh, India; ^3^ Department of Biotechnology, Panjab University, Chandigarh, India

**Keywords:** cancer, nanotechnology, cancer diagnosis, quantum dots, carbon nanodots, bioconjugation

## Abstract

Detection of cancer at an early stage is one of the principal factors associated with successful treatment outcome. However, current diagnostic methods are not capable of making sensitive and robust cancer diagnosis. Nanotechnology based products exhibit unique physical, optical and electrical properties that can be useful in diagnosis. These nanotech-enabled diagnostic representatives have proved to be generally more capable and consistent; as they selectively accumulated in the tumor site due to their miniscule size. This article rotates around the conventional imaging techniques, the use of carbon based nanodots *viz* Carbon Quantum Dots (CQDs), Graphene Quantum Dots (GQDs), Nanodiamonds, Fullerene, and Carbon Nanotubes that have been synthesized in recent years, along with the discovery of a wide range of biomarkers to identify cancer at early stage. Early detection of cancer using nanoconstructs is anticipated to be a distinct reality in the coming years.

## Introduction

Cancer remains among the world’s most devastating diseases with about 20 million cases and 10 million deaths reported as of 2020. The disease is perceived by the condition wherein cells divide uncontrollably and attack different tissues. Most prevalent cancers include breast (11.6%), lung (11.4%), colorectal (10.0%), prostate (7.3%), and stomach cancer (5.6%) (Sung et al., 2021). Although significant progress has been made in diagnosing as well as treating cancer, yet it still accounts for a large number of fatalities.

Imaging techniques like computed tomography (CT), magnetic resonance imaging (MRI), positron emission tomography (PET), and ultrasound are widely used in detecting different cancer types. These techniques are used to locate and visualize cancer but are expensive, need trained staff, cannot be taken to field condition, less sensitive and accurate for early cancer detection, and sometimes involve the use of toxic radiolabeled compounds. Albeit an intrusive biopsy after imaging accompanied by histopathological assessment is the preferred method of diagnosis but this invasive technique requires skilled manpower and is not useful in early cancer diagnosis. Non-obtrusive techniques are still in their infancy, however, of much interest, early cancer diagnosis combined with specific cancer therapies can increase patient survival (Chen et al., 2020). Nanomedicine, a novel research area that blends nanomaterials and medicine, can possibly aid the development of innovative diagnostic tools for detection of primary cancers at initial stages, and for effective cancer therapy (Bar-Zeev et al., 2017).

The possibilities of cancer diagnosis and treatment using nanotechnology are colossal. It has led to the creation of nanomaterials with novel surface architecture and properties, thus opening vast avenues for manipulations at molecular level. Appending antibodies or other targeting agents onto nanocarrier surface for accurately targeting cancer cells is a promising approach for remedial and diagnostic oncology which is bound to take cancer therapy to an altogether different dimension (Farahavar et al., 2019). Nanotechnology is an incredible science not only to modify cancer diagnostics but also to provide detection strategies with higher dependability, sensitivity, and specificity.

Most of the standard chemotherapeutics are non-specific for tumor cells and exhibit toxicity to normal cells in vicinity. In this direction, localizing the drug at the tumor site reduces side effects associated with chemotherapy. Drug delivery systems based on nanotechnology extend the circulation of different chemotherapeutics in blood and improve their solubility (Sharma and Mondal, 2020). The development of biocompatible carbon-based nanomaterials for targeted diagnosis and treatment of diseases is an area of immense interest. This review attempts to give a quick overview of cancer and different imaging techniques used for its detection till date. Further, we aim to highlight emerging applications of nanotechnology, specifically carbon based nanodots for cancer diagnosis along with different bioconjugation techniques employed for this purpose. An extended information is provided in Supplementary file.

## Cancer and Its Pathophysiology

**FIGURE 1:**
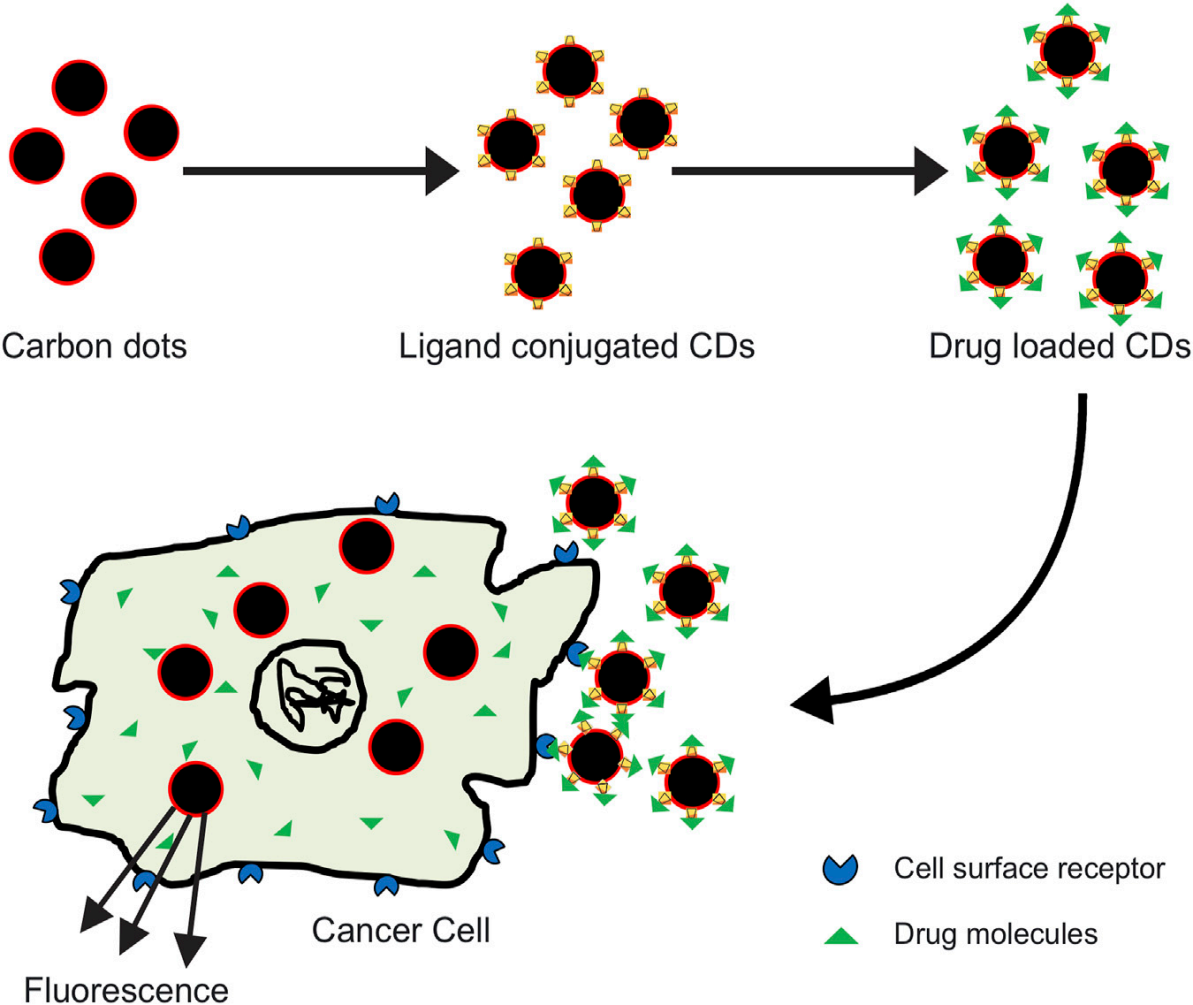
Different processes involved in the progression of malignant neoplasm. Subjecting a normal cell to carcinogenic agents disrupts DNA repair machinery of the cell, inducing DNA damage. This act further triggers activation of oncogenes, inhibition of tumor suppressor genes, and finally leads to apoptosis. These processes altogether contribute to uncontrolled cell differentiation, growth, and ultimately forming malignant neoplasms.

Cancer is a condition involving abnormal division of cells, which invade different tissues. Mutations in genes controlling division of cells gives rise to cancer, which further metastasizes. Typically, human cells grow and divide to create new cells, and therefore the older cells are continually shed and substituted with new cells. But since the lethal disease develops inside the body, this process is hindered. The cells hence become unusual, as damaged cells still survive within the body along with the new cells, while these aren’t actually needed. These additional cells divide ceaselessly, eventually driving tumor spread. The tumor may either be benign or malignant. Benign tumors are not recognized as destructive since they develop gradually and furthermore don’t attack tissues or spread to different parts of the body. On the contrary, malignant tumors spread irrepressibly, resulting in the speedy tumor growth. These tumors in turn attack various parts of the body, through various routes like lymphatic system, blood, and ultimately form new tumors. The human body is composed of trillions of cells, dividing at normal rate and speed. Development of cancer leads to change of normal cells to cancer cells. Cancer cells have different DNA than normal cells, which can trigger extensive damage in the body. The commencement of a cell getting changed into neoplastic cell takes place with change of proto-oncogenes to oncogenes. Proto-oncogenes are significant for typical cell development inside human body. Nevertheless, in contrast, oncogenes cause cell growth to vary and get faster. The second step towards formation of a neoplastic cell is popping off the tumor suppressor genes, which help in preventing cancer from propagating within healthy cells. These genes prevent cell growth, while turning these off ends up in abnormal cell growth and therefore quick division of cells (Figure 1). The last step is turning off of DNA repair genes. These genes are essential for normal functioning of cells and to detect any changes in the DNA. However, turning off these genes makes the cell unable to detect and repair any abnormalities.

## Present Diagnostic Methods

In the battle against cancer, early detection is the key for effective disease treatment. This leads to significant reduction in disease related mortality. Of late, it has become easy to detect and treat cancer because of modern imaging methods and morphological examination of tissues (histopathology) or cells (cytology), which helps in early analysis of malignant growth. Imaging techniques such as X-ray, MRI, CT, endoscopy, and ultrasound can possibly distinguish malignancy when there is a noticeable change in the tissue (Hussain and Nguyen, 2014; García-Figueiras et al., 2019). However, these techniques are unable to differentiate between benign and malignant lesions. These techniques do not permit quantization of the real tumor volume in the specified area. Thus, developing technologies for identification of malignant growth at initial stages is an arduous challenge. Detecting tumors at an early stages is extremely crucial for treatment of cancer. For some cancer types, very few screening tests are available and many of those are not very reliable. Further, non-invasive screening is not available for most of the cancer types and few patients do not adhere to guidelines for screening (Chen et al., 2020).

For accurate cancer detection, nanotechnology based tools with enhanced sensitivity and specificity are being extensively developed (Garrigue et al., 2018). Advances in nanotechnology involve using NPs for non-invasive tumor imaging. Distinctive carbon-based nanomaterials such as CQDs, fullerene, etc. have been in use for cancer diagnosis (Abdolahad et al., 2013; Shi et al., 2014; Kalaiyarasan et al., 2019). A comparative summary of the conventional as well as the relatively new carbon based nanotechnological agents for various cancer diagnoses is provided in Table 1.

**TABLE 1:** A comparative summary of different conventional techniques and carbon-based nanomaterials used in early diagnosis of cancer.

Technique	Principle	Applications	References	Carbon based nanomaterial	Size	Applications	References
Computerized tomography	X-ray beams are used and results are combined to form an image	1. In detecting bone and joint problems	Farwell et al. (2014); Liguori et al. (2015)	Carbon quantum dots	<10 nm	In bioimaging, as biosensors, catalysis, biomedicine delivery system (conjugation of oxidized oxaliplatin with CQDs	Wang and Hu (2014)
		2. For diseases like cancer, emphysema, heart diseases or liver masses					
		3. Visualizing internal injuries, bleeding or hemorrhage					
Positron emission tomography	Radiolabelled tracer molecule administered inside the tissue; depicts image by means of photons/bright spots	1. Neuroimaging	Anand et al. (2009)	Graphene quantum dots	Few nm to ∼100 nm	As photovoltaics, organic light emitting diodes, environment oriented applications, biosensors, cancer bioimaging (i.e., human epithelial cervical cancer)	Bacon et al. (2014)
		2. Clinical oncology					
		3. Musculoskeletal imaging					
		4. Neuropsychology					
		5. Cardiology					
Magnetic resonance imaging	Energy differences between alignment and de-alignment of protons by pre and post magnetic fields are calculated and image is formed	1. Detecting inflammation, vascular abnormalities, and degenerative diseases	Faghihi et al. (2017)	Fullerene	<5 nm	As antioxidants for inflammatory diseases, anti-viral/anti-bacterial agents, diagnostic MRI contrast agents, theranostic for brain cancer	Dellinger et al. (2013)
		2. Detecting gastrointestinal conditions					
Ultrasound imaging	Ultrasound waves get reflected by tissue/organ, thus form the image	1. Detecting changes in internal organs and tissues	Lizzi et al. (2003); Pugash et al. (2008)	Carbon nanotubes	**SWCNT**: 0.4–3 nm diameter and 20–1,000 nm length	As carriers for anti-cancer drugs, for photothermal therapy of cancer, as carrier for immunoactive compounds and genetic material	Elhissi et al. (2012)
		2. Detecting foetal abnormalities			**MWCNT**: 2–100 nm outer diameter 1–3 nm inner diameter and 1- several µm length		

## Nanotechnology for Cancer Diagnosis

The utilization of nanomaterials for clinical diagnostics and drug delivery is gaining importance. Nanotechnology based agents are used in assortment of medical tests and screens for example, the use of gold nanoparticles (AuNPs) for pregnancy test kits (Hartman et al., 2013; Zhou et al., 2015). NPs are also used to detect malignancy biomarkers (Ye et al., 2018; Amri et al., 2021) such as cancer associated proteins, circulating tumor cells (CTCs) and DNA (Wang et al., 2011; Hu et al., 2018), and exosomes (Martín-Gracia et al., 2020). NPs have enormous surface area to volume ratio in comparison to bulk materials. Due to this, surface of nanoparticles can be coated with different moieties like peptides, antibodies, aptamers etc. These moieties can not only bind but also detect cancer molecules. Tunable shape, size and other surface properties additionally impart proper compatibility with different routes of administration, high carrier capacity, and stability, thus rendering them exceptionally alluring for oncological research. These engineered NPs with high contrast bioimaging, fluorescence and carrier functionalities have become admirable tools for molecular diagnostics and delivery of therapeutics. With a reproducible particle size, narrow size distribution and simple synthetic routes; large scale, cost effective products significant for clinical translation can be produced (Bertrand et al., 2014; Chaturvedi et al., 2019). Further information on role of nanotechnology in developing advanced diagnostic methods is mentioned in the Supplementary file.

The following sub-sections tend to highlight various engineered and functionalized carbon-based nanodots carrying a potential role in diagnosis of cancer.

### Carbon Quantum Dots

**FIGURE 2:**
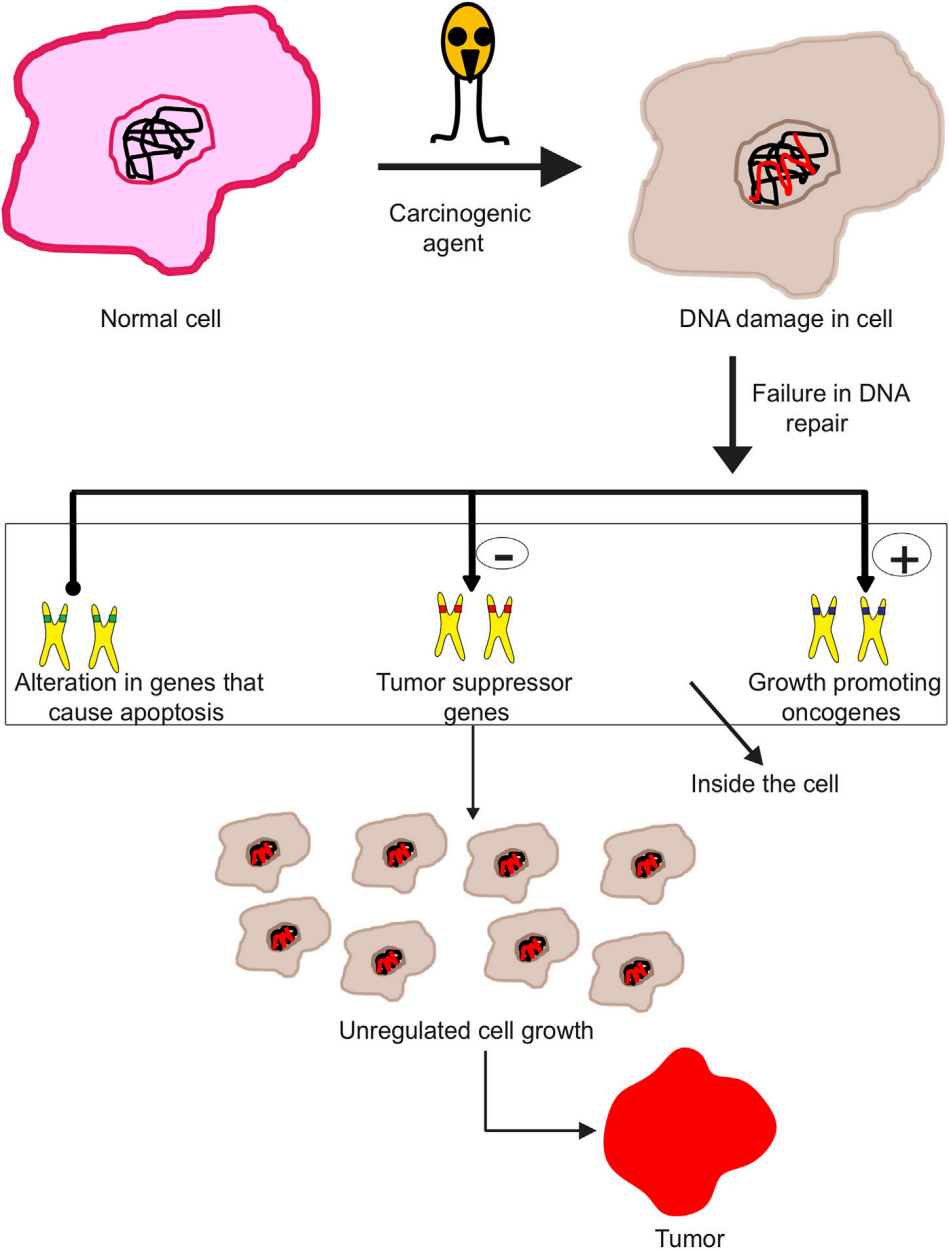
Carbon dots bound to anticancer drug effectively target the tumor cells and deliver the drug molecule at the cell surface; interaction of the drug with tumor cell results in its death.

Since the accidental discovery of carbon dots (CDs), also called carbon quantum dots (CQDs) during separation and purification of single walled carbon nanotubes (SWCNTs), their properties like low toxicity, biocompatibility, fluorescence, and chemical inertness have been utilized in theranostic fields (Figure 2) (Ding et al., 2014; Lim et al., 2015)

Fluorescent CQDs are better than organic dyes in aspects like hydrophilicity, biocompatibility, ease of preparation, and lower toxicity. Due to these reasons, CQDs are considered good for cancer detection. Fluorescent CQDs can be used as imaging probes. Metals like Gadolinium have been used in combination with CQDs, which not only reduce their toxicity to organs but also prevent their leakage (Huang et al., 2020; Jiang et al., 2021). Many fluorescent sensors for Fe^3+^ detection are being developed nowadays, most of them are based on CQDs. Abnormal levels of Fe^3+^ are associated with development of cancer and other diseases. A solution comprising CQDs with doped Fe^3+^ was able to distinguish cancer cells from normal cells majorly because of differences in GSH levels (Gao et al., 2018). CQDs also suppress cancer cells *in vitro* (Pardo et al., 2018; Jia et al., 2020; Su et al., 2020). For visual detection of cancer, green fluorescence CDs and corresponding probe with turn on fluorescence were designed. Cancer cells were imaged by interaction between carbon dots and folic acid. The probe was able to detect folate receptor (FR) positive cells with turn-on fluorescence (Liu et al., 2015). In another study, fluorescent carbon dots conjugated with folic acid that can bind to FR, were prepared. These carbon dots exhibited remarkable biocompatibility and were able to distinguish between normal cells and cancer cells with FR (Song et al., 2012).

### Graphene Quantum Dots

Derived from graphene, graphene quantum dots (GQDs) have good biocompatibility, luminescence and dispersibility in solvents (Younis et al., 2020; Zhao et al., 2020). Because of their intrinsic fluorescence, GQDs are ideal for anti-cancer therapy, permitting effective tracking of cells *in vitro* (Dong et al., 2018; Zhang et al., 2018; Kortel et al., 2020; Iannazzo et al., 2021). GQDs also increase efficiency of anticancer drugs (Fan et al., 2019; Nasrollahi et al., 2019). GQDs are widely used for bioimaging, due to low cytotoxicity and strong optical absorption (Kumar et al., 2020).

GQD based nanomaterials have been effectively used for photodynamic therapy and tumor diagnosis. *In vivo* experiments have revealed that GQDs prevent breast cancer growth in mice (Fan et al., 2019). Nitrogen doped GQDs have been synthesized and used as nanocarriers for delivery of anti-cancer drug methotrexate (Khodadadei et al., 2017). Recently, sulphur doped GQDs conjugated with folic acid have also been synthesized. These GQDs enter FR positive cancer cells via endocytosis and are extremely effective for early cancer diagnosis (Kadian et al., 2020). Due to large surface area to volume ratio, GQDs can support large drug amounts, thus enhancing its delivery. Further, GQDs functionalized with targeted ligands are capable of effectively targeting cancer cells that overexpress particular receptor (Kadian et al., 2021). Recently, biosensors based on “turn on fluorescence” were designed for efficiently detecting biomarkers of lung cancer using combination of GQDs and gold nanoparticles (Kalkal et al., 2020).

### Fullerenes

Fullerenes (C_60_) find application as drug carriers; by means of drug conjugation, fullerene enhances therapeutic effects for paclitaxel-embedded C_60_ (Rezaian et al., 2018) and with doxorubicin (DOX) (Liu et al., 2014; Maleki et al., 2020). Conjugation with fullerenes makes the drug more hydrophilic and less cytotoxic; fullerenes also facilitate delivery of DNA into cells (Nitta et al., 2015; Illescas et al., 2020). In addition, these are also used as photosensitizers and more efficiently in photodynamic therapy; conjugation of C_60_ with poly ethylene glycol (PEG) enhanced their photosensitive effect. Endohedral fullerenes were used to stop angiogenesis and reduce vessel density in tumor tissues. Fullerenes act as immunomodulators and activate various immune cells by generating reactive oxygen species (ROS), thus killing cancer cells. Fullerenes also overcome tumor resistance to chemotherapeutic drugs and its derivatives are used as antioxidant species (Kepinska et al., 2018; Sergeeva et al., 2019). A study involving anti-cancer drug conjugated to C_60_ fullerene showed C_60_ to trigger phagocyte activity. In addition, fullerenes with anticancer drugs promotes ROS production by phagocytes, a promising strategy for treatment of cancer (Skivka et al., 2018).

### Carbon Nanotubes

CNTs are used as contrast agents in medical imaging. They hold several potential advantages over other nano-sized detection agents, such as an exceptionally high surface area and the possibility for incorporating additional therapeutic and diagnostic moieties either on the surface or their inner cavity. The functionalized CNTs have tremendous potential as ultrasound contrast agent, exhibiting support for their future applications as theranostic tools. (Gao, 2018; Gu et al., 2018; Zhang et al., 2019; Zhang et al., 2020a). Photoacoustic imaging through nanotubes facilitates accurate tumor targeting. After conjugation of an MRI active contrast agent, nanotubes efficiently target tumors in magnetic fields. CNTs also facilitate drug delivery to target site and increase blood circulation after successful uptake by cancerous cells (Sanginario et al., 2017). CNTs are also used extensively in photothermal therapy of cancer cells (Lu et al., 2019).

On the basis of diameter and structure, these are further divided into two categories––single walled carbon nanotubes (SWCNTs), with single sheet of graphene in tube form with 0.4–3 nm diameter and multi walled carbon nanotubes (MWCNTs) which comprise of a few layers of graphite with an inner diameter around 1–3 nm and outer diameter between 2 and 100 nm. Like other NPs, CNTs are difficult to dissolve in aqueous medium; hence, these are modified or functionalized, for improved biocompatibility (Chen et al., 2017b).

SWCNTs have applications in delivery of drugs (Kamel et al., 2018), proteins (Antonucci et al., 2017) and siRNA inside target cells (Ravi Kiran et al., 2020). Conjugation of SWCNT with DOX demonstrated better clinical efficacy in comparison to when DOX was used alone. Nanotubes are effective for administering cancer therapy *in vivo* (Mahajan et al., 2018). Another anti-cancer drug, Paclitaxel (PTX) in conjugation with SWCNT effectively suppressed tumors without causing toxicity to other organs, providing evidence that nanotube based drug delivery is favorable for cancer therapy due to high treatment efficiency and low toxicity (Yu et al., 2016; Hashemzadeh and Raissi, 2017). MWCNTs were used for developing magnetic nanocarrier using iron oxide NPs. MWCNTs thus developed showed dual targeted delivery (Boncel et al., 2017; Fan et al., 2018; Hossen et al., 2019). A new method for delivering DNA and siRNA into microglia for brain cancer therapy via MWCNTs has been devised (Xiang et al., 2020).

### Nanodiamonds

Nanodiamonds refer to a colloidal suspension of diamond particles. The use of these fluorescent nanodiamonds has started extensively, especially for bioimaging due to their low toxicity in comparison to quantum dots (Wei et al., 2019; Lai et al., 2020; Reineck et al., 2021). Nanodiamonds coupled to fluorophores are capable of targeting tumor cells without affecting cell viability and getting degraded due to changing pH (Yu et al., 2019; Liu et al., 2020; Perevedentseva et al., 2020).

Even the poorly soluble drugs can be adsorbed on the surface of nanodiamonds, which then mediate their slow and sustained release (Chipaux et al., 2018). The nanodiamonds based drug delivery systems are associated with reduced resistance to chemotherapeutics in different cancer types. DOX, considered effective for many types of cancer has been successfully adsorbed on nanodiamonds (called NDX). The resulting NDX particles were found to be taken up by living cells rapidly, facilitating drug delivery inside the cells. Another drug, epirubicin when adsorbed on nanodiamonds, leads to improved retention of tumor cells thereby killing cancer as well as non-cancer stem cells both *in vivo* and *in vitro* conditions. This prevents the formation of secondary tumors (Lai et al., 2020). Nanodiamonds have been used as vector for delivery of siRNA on sarcoma cells (Claveau et al., 2020). Detonation Nanodiamonds (DNDs) have been reported to be useful in radiotherapy. Irradiation of cancer cells resistant to radiotherapy is more effective when DNDs are incorporated inside the cells (Matshitse et al., 2020). For tumor therapy, delivery of sodium ions inside the cell has been easily carried out with the help of nanodiamonds (Chen et al., 2017a; Gupta et al., 2017). Nanodiamonds have been successfully used to target autophagy in tumor cells and promote their programmed cell death (PCD) in hypoxic conditions *in vivo* (Chen et al., 2018).

### Nanocantilevers

Nanocantilevers can be coated with substrates that can selectively bind the target and detect even minute molecules in biological fluids. Binding of nanocantilevers to biomolecules changes the baseline probe frequency. These differences in frequency are measured by light diffraction pattern or by electrical means (Jabbari Behrouz et al., 2019). As the target sequence binds, the signal is transduced mechanically to surface of cantilever, resulting in its bending. Detection of cancer molecules can be done on the basis of deflection, which depends on the amount of DNA bound to cantilever surface and can be observed (Wang et al., 2016; Haring et al., 2017). This technology has been successfully used for differentiating between *BRAF* and its wild type gene in melanoma patients (Huber et al., 2013). The elevated levels of prostate specific antigen (PSA) are associated with increased risk of prostate cancer among men. Using antibodies bound to the surface of cantilevers, PSA assay was performed (Damborska et al., 2017; Basu et al., 2020).

## Bioconjugation Strategies for Nanotechnology-Based Agents

The surface of nanodots must be modified for compatibility with biological systems in order to facilitate their *in vitro* and *in vivo* applications. Surface functionalization improves stability and water-solubility, of nanodots, which can be further conjugated with biomolecules of interest for biomedical applications (Valcourt et al., 2018; Xu et al., 2020). Different techniques involving coupling are in use for a long time. There are two main strategies for conjugation of biomolecules: covalent and non-covalent binding. Non-covalent binding further involves coupling through direct absorption and electrostatic interactions (Foubert et al., 2016). As the name implies, direct absorption approach involves direct interaction of biomolecules with various NPs. Cell structures can be nonspecifically stained using hydrophilic NPs. Usually, the interaction is nonspecific and weak. Biomolecules with particular functional groups can be directly attached to NPs, e.g., thiol groups from cytosine can bind proteins to noble metal NPs. Another methodology is to absorb biomolecules on surface of NPs through electrostatic interaction, e.g., positively charged NPs can be attached to negatively charged nucleic acid (Sapsford et al., 2013; Boehnke et al., 2020).

Covalent coupling involves precise and stable conjugation of biomolecules with NPs. Generally, coupling reactions to crosslink biomolecules are carried out on the surface of NPs using functional groups like carboxylic, amine, and thiol group. Carboxyl group coupling includes reaction of primary amines with carboxylic group. The coupling agent used is 1-ethyl-3-(3-dimethylaminopropyl)carbodiimide (EDC). Additionally, stabilizing agents like n-hydroxysuccinimide (NHS) or sulfo-NHS enhance coupling efficiency by arrangement of a succinimide ester intermediate (Blanco-Canosa et al., 2014; Karakoti et al., 2015; Saallah and Lenggoro, 2018). Amine group coupling involves reaction of carboxylic group with amine group for forming an amide bond. The coupling reagent used is glutaraldehyde, which activates NH_2_-functionalized NPs. The aldehyde group produced throughout actuation will conjugate with amino groups of biomolecules (Barbosa et al., 2014; Zhang et al., 2015). Thiol group coupling involves conjugation of thiol group with primary amine groups. The reaction begins quickly using reagents like maleimides and iodoacetamides. sulfosuccinimidyl4-(maleimidomethyl) cyclohexane-1-carboxylate (sulfo-SMCC) is used for coupling. Amine-functionalized NPs can be conjugated to various thiolated biomolecules, like residue thiol-terminated DNA, thiol-modified peptide, or peptides, and proteins with free or reduced cysteine (Brodie et al., 2015; Karakoti et al., 2015; Kolodych et al., 2015; Martinez-Jothar et al., 2018) (Various bioconjugation strategies are depicted in Supplementary Figure S1)

## Cancer Biomarkers

Cancer markers assist detection of specific types of cancer in the body and in noticing the progression of cancer treatment. Cancer biomarkers register their presence in body fluids, blood or tissues that aid in detection of cancer cells and assist in setting up specific diagnosis. This is particularly the circumstance when there is a need to decide if the tumors are primary or metastatic in origin. Many biomarkers specific to different cancer types have been discovered till date. These have crucial roles for early diagnosis of cancer. A description of some such biomarkers for different cancers is given in Table 2 along with an assortment of carbon nanomaterials used for the detection and/or treatment of cancer types. Detailed information on cancer biomarkers that aid the diagnosis of different cancer types is provided in Supplementary file.

**TABLE 2:** Chart of different biomarkers and carbon based nanomaterials for cancer diagnosis and treatment.

Cancer type	Biomarkers	References	Carbon-based nanomaterial for detection and/or treatment
Bladder	Nuclear matrix protein 22 (NMP-22), BTA Stat and BTA-TRAK, UroVysion, ImmunoCyt/uCyt+, Uromonitor and Uromonitor-V2, UroSEEK, EpiCheck, TERTp mutations and hypermethylation	Batista et al. (2020)	•Graphene quantum dots Deng et al. (2020) •Nitrogen doped carbon dots Othman et al. (2020)
Breast	**Conventional and non-conventional markers:** Estrogen receptor (ER), progesterone receptor (PR), nuclear antigen Ki-67, human epidermal growth factor receptor (HER) 2 MammaPrint®, Oncotype DX®, Prosigna®, EndoPredict®	Colomer et al. (2018)	•Carbon dots from N-hydroxyphthalimide (CD-NHF) Tiron et al. (2020) •Fluorescent carbon nanodots García-Mendiola et al. (2019)
Cervical	Human papillomavirus DNA (HPV DNA), squamous cell carcinoma antigen (SCC-Ag), serum fragments of cytokeratin (CYFRA), carcinoma embryonic antigen (CEA), soluble CD44 (Scd44)	Kori and Yalcin Arga (2018)	•Carbon dots embedded molecularly imprinted polymers (C-MIP) Zhang et al. (2020b) •Graphene oxide-silver nanoparticle conjugate Yuan and Gurunathan (2017)
Colorectal	**Tissue-based biomarkers**: serine/threonine protein kinase (encoded by BRAF gene), kirsten rat sarcoma viral oncogene homolog (KRAS), microsatellite instability (MSI-H+) **Epigenetic markers:** CpG island methylator phenotype (CIMP), adenomatous polyposis *coli* (APC) **Other promising biomarkers:** Phosphatidylinositide-3-kinases, phosphatase and tensin homologue (PTEN), tumor protein p53 (TP53), NDST4, chromosome 18q loss of heterozygosity (LOH), insulin-like growth factor type 1 receptor (IGFR-1R)	Vacante et al. (2018)	•Graphene oxide, carbon nano-onions Ibáñez-Redín et al. (2019) •Hyaluronate functionalized graphene (HG) Jing et al. (2018)
Liver	Alpha-fetoprotein (AFP) L3, des-γ-carboxyprothrombin, glypican-3, cytokeratin 19, golgi protein-73, midkine, osteopontin, SCC-Ag, annexin A2, circulating microRNAs, cell-free DNA	Parikh et al. (2020)	•Multi-wall carbon nanotube loaded with sorafenib Elsayed et al. (2019) •Graphene quantum dots Li et al. (2018)
Lung	**Genomic biomarkers**: Epidermal growth factor receptor (EGFR), Anaplastic lymphoma kinase (ALK), (KRAS), ROS proto-oncogene 1, receptor tyrosine kinase (ROS1), HER2, RET proto-oncogene, MET proto-oncogene, BRAF proto-oncogene, BRAF, Phosphatidylinositol-4,5-bisphosphate 3-kinase catalytic subunit alpha (PIK3CA), Neurotrophic receptor tyrosine kinase 1 (NTRK1), Fibroblast growth factor receptor (FGFR), Discoidin domain receptor tyrosine kinase 2 (DDR2) **Immunotherapy markers in lung cancer**: Cytotoxic T-lymphocyte-associated antigen 4 (CTLA-4), Programmed death-ligand 1 receptor (PD-1)	Villalobos and Wistuba (2017)	•Single-wall carbon nanotubes Aasi et al. (2020) •Multi-wall carbon nanotubes Janfaza et al. (2019) •Graphene quantum dots and gold nanoparticles Kalkal et al. (2020)
Oesophagus	p75 neurotrophin receptor (NTR) (CD271), CD44, aldehyde dehydrogenase (ALDH), CD90, intercellular adhesion molecule 1 (ICAM1), Cripto-1, ALDH1A1, CD133, CXCR4, ABCG2	Liu et al. (2019)	•Hollow carbon spheres Zhang et al. (2017) •Graphene oxide Jiang et al. (2018)
Prostate	Aberrant serum PSA glycosylation (S2, 3PSA), progensa PCA3 assay, transmembrane protease serine 2 (TMPRSS2)- erythroblastosis virus E26 oncogene homolog (ERG) fusion gene, Mi-prostate score (MiPS), oncotype DX test, prolaris test, decipher genomic classifier (decipher GC), ProMark, Core 2 β-1,6-N-acetylglucosaminyltransferase-1 (GCNT1), circulating tumor cells (CTCs), urokinase plasminogen activator (uPA)	Hatakeyama et al. (2017)	•Single wall carbon nanotubes Williams et al. (2018) •Multi-wall carbon nanotubes with gold nanoparticles Quintero-Jaime et al. (2019)
Stomach	Carcinoembryonic antigen (CEA), cancer antigen 19-9 (CA 19-9), CA72-4, AFP, CA125, HER2	Matsuoka and Yashiro (2018)	•Nanodiamonds loaded with doxorubicin Qin et al. (2019) •Carbon nanoparticles Yan et al. (2016)
Thyroid	Thyroid peroxidase, calcitonin, cytokeratin-19, hector battifora mesothelial antigen, galectin-3, Cbp/p300-interacting transactivator with Glu/Asp-rich carboxy-terminal domain 1 (CITED-1), hepatocyte growth factor, epidermal growth factor	Arcolia et al. (2017); Xiao et al. (2020)	•Carbon nanoparticles Liu et al. (2018) •Multi-wall carbon nanotubes Dotan et al. (2016)

## Conclusion

Cancer nanotechnology holds promise in providing novel techniques for cancer detection at initial stages, resulting in improved diagnosis, and treatment. Conventional imaging techniques are exceptionally intrusive, non-specific, and are frequently associated with toxicity to both tumor and solid cells. The advancement of novel nanomaterials has enabled the identification of cancer biomarkers with more sensitivity and accuracy that was not feasible previously. Steady and coordinated exploration endeavors should be embraced to utilize tremendous capability of nanotechnology in distinguishing cancer growth in early stages, and monitoring the disease with treatment precision.

## References

[B1] Aasi A. Aghaei S. M. Panchapakesan B. (2020). A Density Functional Theory Study on the Interaction of Toluene with Transition Metal Decorated Carbon Nanotubes: a Promising Platform for Early Detection of Lung Cancer from Human Breath. Nanotechnology 31, 415707. 10.1088/1361-6528/ab9da9 32554899

[B2] Abdolahad M. Janmaleki M. Taghinejad M. Taghnejad H. Salehi F. Mohajerzadeh S. (2013). Single-cell Resolution Diagnosis of Cancer Cells by Carbon Nanotube Electrical Spectroscopy. Nanoscale 5, 3421–3427. 10.1039/c3nr33430a 23474499

[B3] Amri C. Shukla A. K. Lee J. H. (2021). Recent Advancements in Nanoparticle-Based Optical Biosensors for Circulating Cancer Biomarkers. Materials (Basel) 14. 10.3390/ma14061339 PMC8001438 33802028

[B4] Anand S. Singh H. Dash A. (2009). Clinical Applications of PET and PET-CT. Med. J. Armed Forces India 65, 353–358. 10.1016/s0377-1237(09)80099-3 27408291 PMC4921358

[B5] Antonucci A. Kupis-Rozmysłowicz J. Boghossian A. A. (2017). Noncovalent Protein and Peptide Functionalization of Single-Walled Carbon Nanotubes for Biodelivery and Optical Sensing Applications. ACS Appl. Mater. Inter. 9, 11321–11331. 10.1021/acsami.7b00810 28299937

[B6] Arcolia V. Journe F. Renaud F. Leteurtre E. Gabius H.-J. Remmelink M. (2017). Combination of Galectin-3, CK19 and HBME-1 Immunostaining Improves the Diagnosis of Thyroid Cancer. Oncol. Lett. 14, 4183–4189. 10.3892/ol.2017.6719 28943926 PMC5592881

[B7] Bacon M. Bradley S. J. Nann T. (2014). Graphene Quantum Dots. Part. Part. Syst. Charact. 31, 415–428. 10.1002/ppsc.201300252

[B8] Bar-Zeev M. Livney Y. D. Assaraf Y. G. (2017). Targeted Nanomedicine for Cancer Therapeutics: Towards Precision Medicine Overcoming Drug Resistance. Drug Resist. Updates 31, 15–30. 10.1016/j.drup.2017.05.002 28867241

[B9] Barbosa O. Ortiz C. Berenguer-Murcia Á. Torres R. Rodrigues R. C. Fernandez-Lafuente R. (2014). Glutaraldehyde in Bio-Catalysts Design: a Useful Crosslinker and a Versatile Tool in Enzyme Immobilization. RSC Adv. 4, 1583–1600. 10.1039/c3ra45991h

[B10] Basu A. K. Basu A. Bhattacharya S. (2020). Micro/Nano Fabricated Cantilever Based Biosensor Platform: A Review and Recent Progress. Enzyme Microb. Tech. 139, 109558. 10.1016/j.enzmictec.2020.109558 32732024

[B11] Batista R. Vinagre N. Meireles S. Vinagre J. Prazeres H. Leão R. (2020). Biomarkers for Bladder Cancer Diagnosis and Surveillance: A Comprehensive Review. Diagnostics (Basel) 10. 10.3390/diagnostics10010039 PMC7169395 31941070

[B12] Bertrand N. Wu J. Xu X. Kamaly N. Farokhzad O. C. (2014). Cancer Nanotechnology: the Impact of Passive and Active Targeting in the Era of Modern Cancer Biology. Adv. Drug Deliv. Rev. 66, 2–25. 10.1016/j.addr.2013.11.009 24270007 PMC4219254

[B13] Blanco-Canosa J. B. Wu M. Susumu K. Petryayeva E. Jennings T. L. Dawson P. E. (2014). Recent Progress in the Bioconjugation of Quantum Dots. Coord. Chem. Rev. 263-264, 101–137. 10.1016/j.ccr.2013.08.030

[B14] Boehnke N. Dolph K. J. Juarez V. M. Lanoha J. M. Hammond P. T. (2020). Electrostatic Conjugation of Nanoparticle Surfaces with Functional Peptide Motifs. Bioconjug. Chem. 31, 2211–2219. 10.1021/acs.bioconjchem.0c00384 32786506 PMC7895459

[B15] Boncel S. Pluta A. Skonieczna M. Gondela A. Maciejewska B. Herman A. P. (2017). Hybrids of Iron-Filled Multiwall Carbon Nanotubes and Anticancer Agents as Potential Magnetic Drug Delivery Systems: *In Vitro* Studies against Human Melanoma, Colon Carcinoma, and Colon Adenocarcinoma. J. Nanomater., 2017 **,** 1–13. 10.1155/2017/1262309

[B16] Brodie N. I. Makepeace K. A. T. Petrotchenko E. V. Borchers C. H. (2015). Isotopically-coded Short-Range Hetero-Bifunctional Photo-Reactive Crosslinkers for Studying Protein Structure. J. Proteomics 118, 12–20. 10.1016/j.jprot.2014.08.012 25192908

[B17] Chaturvedi V. K. Singh A. Singh V. K. Singh M. P. (2019). Cancer Nanotechnology: A New Revolution for Cancer Diagnosis and Therapy. Curr. Drug. Metab. 20, 416–429. 10.2174/1389200219666180918111528 30227814

[B19] Chen C. Cho I. C. Jian H. S. Niu H. (2017a). Fe Doped Magnetic Nanodiamonds Made by Ion Implantation. Sci. Rep. 7, 41938. 10.1038/srep41938 28181507 PMC5299451

[B21] Chen N. Han Y. Luo Y. Zhou Y. Hu X. Yu Y. (2018). Nanodiamond-based Non-canonical Autophagy Inhibitor Synergistically Induces Cell Death in Oxygen-Deprived Tumors. Mater. Horiz. 5, 1204–1210. 10.1039/c8mh00993g

[B22] Chen X. Gole J. Gore A. He Q. Lu M. Min J. (2020). Non-invasive Early Detection of Cancer Four Years before Conventional Diagnosis Using a Blood Test. Nat. Commun. 11, 3475. 10.1038/s41467-020-17316-z 32694610 PMC7374162

[B23] Chen Z. Zhang A. Wang X. Zhu J. Fan Y. Yu H. (2017b). The Advances of Carbon Nanotubes in Cancer Diagnostics and Therapeutics. J. Nanomater. 2017, 3418932 10.1155/2017/3418932.

[B25] Chipaux M. Van Der Laan K. J. Hemelaar S. R. Hasani M. Zheng T. Schirhagl R. (2018). Nanodiamonds and Their Applications in Cells. Small 14, 1704263. 10.1002/smll.201704263 29573338

[B26] Claveau S. Nehlig É. Garcia-Argote S. Feuillastre S. Pieters G. Girard H. A. (2020). Delivery of siRNA to Ewing Sarcoma Tumor Xenografted on Mice, Using Hydrogenated Detonation Nanodiamonds: Treatment Efficacy and Tissue Distribution. Nanomaterials 10, 553. 10.3390/nano10030553 PMC7153391 32204428

[B27] Colomer R. Aranda-López I. Albanell J. García-Caballero T. Ciruelos E. López-García M. Á. (2018). Biomarkers in Breast Cancer: A Consensus Statement by the Spanish Society of Medical Oncology and the Spanish Society of Pathology. Clin. Transl Oncol. 20, 815–826. 10.1007/s12094-017-1800-5 29273958 PMC5996012

[B28] Damborska D. Bertok T. Dosekova E. Holazova A. Lorencova L. Kasak P. (2017). Nanomaterial-based Biosensors for Detection of Prostate Specific Antigen. Microchim Acta 184, 3049–3067. 10.1007/s00604-017-2410-1 PMC5669453 29109592

[B29] Dellinger A. Zhou Z. Connor J. Madhankumar A. Pamujula S. Sayes C. M. (2013). Application of Fullerenes in Nanomedicine: an Update. Nanomedicine 8, 1191–1208. 10.2217/nnm.13.99 23837857

[B30] Deng M. Cao X. Guo L. Cao H. Wen Z. Mao C. (2020). Graphene Quantum Dots: Efficient Mechanosynthesis, white-light and Broad Linear Excitation-dependent Photoluminescence and Growth Inhibition of Bladder Cancer Cells. Dalton Trans. 49, 2308–2316. 10.1039/c9dt04575a 32016190

[B31] Ding C. Zhu A. Tian Y. (2014). Functional Surface Engineering of C-Dots for Fluorescent Biosensing and *In Vivo* Bioimaging. Acc. Chem. Res. 47, 20–30. 10.1021/ar400023s 23911118

[B32] Dong J. Wang K. Sun L. Sun B. Yang M. Chen H. (2018). Application of Graphene Quantum Dots for Simultaneous Fluorescence Imaging and Tumor-Targeted Drug Delivery. Sensors Actuators B: Chem. 256, 616–623. 10.1016/j.snb.2017.09.200

[B33] Dotan I. Roche P. J. R. Paliouras M. Mitmaker E. J. Trifiro M. A. (2016). Engineering Multi-Walled Carbon Nanotube Therapeutic Bionanofluids to Selectively Target Papillary Thyroid Cancer Cells. PloS one 11–e0149723., 10.1371/journal.pone.0149723 PMC4762941 26901566

[B34] Elhissi A. M. Ahmed W. Hassan I. U. Dhanak V. R. D'emanuele A. (2012). Carbon Nanotubes in Cancer Therapy and Drug Delivery. J. Drug Deliv. 2012, 837327. 10.1155/2012/837327 22028974 PMC3199121

[B35] Elsayed M. M. A. Mostafa M. E. Alaaeldin E. Sarhan H. A. A. Shaykoon M. S. A. Allam S. (2019). Design and Characterisation of Novel Sorafenib-Loaded Carbon Nanotubes with Distinct Tumour-Suppressive Activity in Hepatocellular Carcinoma. Int. J. Nanomedicine. Vol. 14, 8445–8467. 10.2147/ijn.s223920 PMC6825507 31754301

[B36] Faghihi R. Zeinali-Rafsanjani B. Mosleh-Shirazi M.-A. Saeedi-Moghadam M. Lotfi M. Jalli R. (2017). Magnetic Resonance Spectroscopy and its Clinical Applications: A Review. J. Med. Imaging Radiat. Sci. 48, 233–253. 10.1016/j.jmir.2017.06.004 31047406

[B37] Fan H.-Y. Yu X.-H. Wang K. Yin Y.-J. Tang Y.-J. Tang Y.-L. (2019). Graphene Quantum Dots (GQDs)-Based Nanomaterials for Improving Photodynamic Therapy in Cancer Treatment. Eur. J. Med. Chem. 182, 111620. 10.1016/j.ejmech.2019.111620 31470307

[B38] Fan H. Duan P. Guo S. Zhao L. Wang K. Qiu D. (2018). Multifunctional MWCNTs@CoFe2O4@mSiO2@NaYF4:Yb3+, Er3+ Nanocomposites and Their Application as Drug Carrier. Mater. Lett. 213, 311–314. 10.1016/j.matlet.2017.11.098

[B39] Farahavar G. Abolmaali S. S. Gholijani N. Nejatollahi F. (2019). Antibody-guided Nanomedicines as Novel Breakthrough Therapeutic, Diagnostic and Theranostic Tools. Biomater. Sci. 7, 4000–4016. 10.1039/c9bm00931k 31355391

[B40] Farwell M. D. Pryma D. A. Mankoff D. A. (2014). PET/CT Imaging in Cancer: Current Applications and Future Directions. Cancer 120, 3433–3445. 10.1002/cncr.28860 24947987

[B41] Foubert A. Beloglazova N. V. Rajkovic A. Sas B. Madder A. Goryacheva I. Y. (2016). Bioconjugation of Quantum Dots: Review & Impact on Future Application. Trac Trends Anal. Chem. 83, 31–48. 10.1016/j.trac.2016.07.008

[B42] Gao G. Jiang Y.-W. Jia H.-R. Yang J. Wu F.-G. (2018). On-off-on Fluorescent Nanosensor for Fe3+ Detection and Cancer/normal Cell Differentiation via Silicon-Doped Carbon Quantum Dots. Carbon 134, 232–243. 10.1016/j.carbon.2018.02.063

[B43] Gao Y. (2018). Carbon Nano-Allotrope/Magnetic Nanoparticle Hybrid Nanomaterials as T2 Contrast Agents for Magnetic Resonance Imaging Applications. J. Funct. Biomater. 9. 16. 10.3390/jfb9010016 PMC5872102 29415438

[B44] García-Figueiras R. Baleato-González S. Padhani A. R. Luna-Alcalá A. Vallejo-Casas J. A. Sala E. (2019). How Clinical Imaging Can Assess Cancer Biology. Insights. Imaging. 10, 28. 10.1186/s13244-019-0703-0 30830470 PMC6399375

[B45] García-Mendiola T. Elosegui C. G. Bravo I. Pariente F. Jacobo-Martin A. Navio C. (2019). Fluorescent C-NanoDots for Rapid Detection of BRCA1, CFTR and MRP3 Gene Mutations. Microchimica Acta 186, 293. 10.1007/s00604-019-3386-9 31016506

[B46] Garrigue P. Tang J. Ding L. Bouhlel A. Tintaru A. Laurini E. (2018). Self-assembling Supramolecular Dendrimer Nanosystem for PET Imaging of Tumors. Proc. Natl. Acad. Sci. USA 115, 11454–11459. 10.1073/pnas.1812938115 30348798 PMC6233080

[B47] Gu F. Hu C. Xia Q. Gong C. Gao S. Chen Z. (2018). Aptamer-conjugated Multi-Walled Carbon Nanotubes as a New Targeted Ultrasound Contrast Agent for the Diagnosis of Prostate Cancer. J. Nanopart Res. 20, 303. 10.1007/s11051-018-4407-z 30524190 PMC6244773

[B48] Gupta S. Evans B. Henson A. Carrizosa S. B. (2017). Salt-Assisted Ultrasonicated De-aggregation and Advanced Redox Electrochemistry of Detonation Nanodiamond. Materials (Basel) 10. 10.3390/ma10111292 PMC5706239 29125547

[B49] Haring A. P. Cesewski E. Johnson B. N. (2017). Piezoelectric Cantilever Biosensors for Label-free, Real-Time Detection of DNA and RNA. Methods Mol. Biol. 1572, 247–262. 10.1007/978-1-4939-6911-1_17 28299693

[B50] Hartman M. R. Ruiz R. C. H. Hamada S. Xu C. Yancey K. G. Yu Y. (2013). Point-of-care Nucleic Acid Detection Using Nanotechnology. Nanoscale 5, 10141–10154. 10.1039/c3nr04015a 24057263

[B51] Hashemzadeh H. Raissi H. (2017). The Functionalization of Carbon Nanotubes to Enhance the Efficacy of the Anticancer Drug Paclitaxel: a Molecular Dynamics Simulation Study. J. Mol. Model. 23, 222. 10.1007/s00894-017-3391-z 28702805

[B52] Hatakeyama S. Yoneyama T. Tobisawa Y. Ohyama C. (2017). Recent Progress and Perspectives on Prostate Cancer Biomarkers. Int. J. Clin. Oncol. 22, 214–221. 10.1007/s10147-016-1049-y 27730440 PMC5378754

[B54] Hossen S. Hossain M. K. Basher M. K. Mia M. N. H. Rahman M. T. Uddin M. J. (2019). Smart Nanocarrier-Based Drug Delivery Systems for Cancer Therapy and Toxicity Studies: A Review. J. Adv. Res. 15, 1–18. 10.1016/j.jare.2018.06.005 30581608 PMC6300464

[B55] Hu P. Zhang S. Wu T. Ni D. Fan W. Zhu Y. (2018). Fe-Au Nanoparticle-Coupling for Ultrasensitive Detections of Circulating Tumor DNA. Adv. Mater. 30, 1801690. 10.1002/adma.201801690 29931715

[B56] Huang Y. Li L. Zhang D. Gan L. Zhao P. Zhang Y. (2020). Gadolinium-doped Carbon Quantum Dots Loaded Magnetite Nanoparticles as a Bimodal Nanoprobe for Both Fluorescence and Magnetic Resonance Imaging. Magn. Reson. Imaging 68, 113–120. 10.1016/j.mri.2020.02.003 32032662

[B57] Huber F. Lang H. P. Backmann N. Rimoldi D. Gerber C. (2013). Direct Detection of a BRAF Mutation in Total RNA from Melanoma Cells Using Cantilever Arrays. Nat. Nanotech 8, 125–129. 10.1038/nnano.2012.263 23377457

[B58] Hussain T. Nguyen Q. T. (2014). Molecular Imaging for Cancer Diagnosis and Surgery. Adv. Drug Deliv. Rev. 66, 90–100. 10.1016/j.addr.2013.09.007 24064465 PMC4464660

[B59] Iannazzo D. Celesti C. Espro C. (2021). Recent Advances on Graphene Quantum Dots as Multifunctional Nanoplatforms for Cancer Treatment. Biotechnol. J. 16, e1900422. 10.1002/biot.201900422 32618417

[B60] Ibáñez-Redín G. Furuta R. H. M. Wilson D. Shimizu F. M. Materon E. M. Arantes L. M. R. B. (2019). Screen-printed Interdigitated Electrodes Modified with Nanostructured Carbon Nano-Onion Films for Detecting the Cancer Biomarker CA19-9. Mater. Sci. Eng. C 99, 1502–1508. 10.1016/j.msec.2019.02.065 30889686

[B61] Illescas B. M. Pérez-Sánchez A. Mallo A. Martín-Domenech Á. Rodríguez-Crespo I. Martín N. (2020). Multivalent Cationic Dendrofullerenes for Gene Transfer: Synthesis and DNA Complexation. J. Mater. Chem. B 8, 4505–4515. 10.1039/d0tb00113a 32369088

[B62] Jabbari Behrouz S. Rahmani O. Hosseini S. A. (2019). On Nonlinear Forced Vibration of Nano Cantilever-Based Biosensor via Couple Stress Theory. Mech. Syst. Signal Process. 128, 19–36. 10.1016/j.ymssp.2019.03.020

[B63] Janfaza S. Banan Nojavani M. Nikkhah M. Alizadeh T. Esfandiar A. Ganjali M. R. (2019). A Selective Chemiresistive Sensor for the Cancer-Related Volatile Organic Compound Hexanal by Using Molecularly Imprinted Polymers and Multiwalled Carbon Nanotubes. Microchimica Acta 186, 137. 10.1007/s00604-019-3241-z 30707323

[B64] Jia Q. Zhao Z. Liang K. Nan F. Li Y. Wang J. (2020). Recent Advances and Prospects of Carbon Dots in Cancer Nanotheranostics. Mater. Chem. Front. 4, 449–471. 10.1039/c9qm00667b

[B65] Jiang J.-H. Pi J. Jin H. Cai J.-Y. (2018). Functional Graphene Oxide as Cancer-Targeted Drug Delivery System to Selectively Induce Oesophageal Cancer Cell Apoptosis. Artif. Cell Nanomedicine, Biotechnol. 46, S297–S307. 10.1080/21691401.2018.1492418 30183382

[B66] Jiang Q. Liu L. Li Q. Cao Y. Chen D. Du Q. (2021). NIR-laser-triggered Gadolinium-Doped Carbon Dots for Magnetic Resonance Imaging, Drug Delivery and Combined Photothermal Chemotherapy for Triple Negative Breast Cancer. J. Nanobiotechnology 19, 64. 10.1186/s12951-021-00811-w 33653352 PMC7923633

[B67] Jing A. Zhang C. Liang G. Feng W. Tian Z. Jing C. (2018). Hyaluronate-Functionalized Graphene for Label-free Electrochemical Cytosensing. Micromachines 9, 669. 10.3390/mi9120669 PMC6315524 30567299

[B68] Kadian S. Manik G. Das N. Roy P. (2020). Targeted Bioimaging and Sensing of Folate Receptor-Positive Cancer Cells Using Folic Acid-Conjugated Sulfur-Doped Graphene Quantum Dots. Microchimica Acta 187, 458. 10.1007/s00604-020-04448-8 32683509

[B69] Kadian S. Sethi S. K. Manik G. (2021). Recent Advancements in Synthesis and Property Control of Graphene Quantum Dots for Biomedical and Optoelectronic Applications. Mater. Chem. Front. 5, 627–658. 10.1039/d0qm00550a

[B70] Kalaiyarasan G. Veerapandian M. Jebamercy G. Balamurugan K. Joseph J. (2019). Amygdalin-Functionalized Carbon Quantum Dots for Probing β-Glucosidase Activity for Cancer Diagnosis and Therapeutics. ACS Biomater. Sci. Eng. 5, 3089–3099. 10.1021/acsbiomaterials.9b00394 33405541

[B71] Kalkal A. Pradhan R. Kadian S. Manik G. Packirisamy G. (2020). Biofunctionalized Graphene Quantum Dots Based Fluorescent Biosensor toward Efficient Detection of Small Cell Lung Cancer. ACS Appl. Bio Mater. 3, 4922–4932. 10.1021/acsabm.0c00427 35021736

[B72] Kamel M. Raissi H. Morsali A. Shahabi M. (2018). Assessment of the Adsorption Mechanism of Flutamide Anticancer Drug on the Functionalized Single-Walled Carbon Nanotube Surface as a Drug Delivery Vehicle: An Alternative Theoretical Approach Based on DFT and MD. Appl. Surf. Sci. 434, 492–503. 10.1016/j.apsusc.2017.10.165

[B73] Karakoti A. S. Shukla R. Shanker R. Singh S. (2015). Surface Functionalization of Quantum Dots for Biological Applications. Adv. Colloid Interf. Sci. 215, 28–45. 10.1016/j.cis.2014.11.004 25467038

[B74] Kepinska M. Kizek R. Milnerowicz H. (2018). Metallothionein and Superoxide Dismutase-Antioxidative Protein Status in Fullerene-Doxorubicin Delivery to MCF-7 Human Breast Cancer Cells. Int. J. Mol. Sci. 19. 10.3390/ijms19103253 PMC6214080 30347787

[B75] Khodadadei F. Safarian S. Ghanbari N. (2017). Methotrexate-loaded Nitrogen-Doped Graphene Quantum Dots Nanocarriers as an Efficient Anticancer Drug Delivery System. Mater. Sci. Eng. C 79, 280–285. 10.1016/j.msec.2017.05.049 28629019

[B76] Kolodych S. Koniev O. Baatarkhuu Z. Bonnefoy J.-Y. Debaene F. Cianférani S. (2015). CBTF: New Amine-To-Thiol Coupling Reagent for Preparation of Antibody Conjugates with Increased Plasma Stability. Bioconjug. Chem. 26, 197–200. 10.1021/bc500610g 25614935

[B77] Kori M. Yalcin Arga K. (2018). Potential Biomarkers and Therapeutic Targets in Cervical Cancer: Insights from the Meta-Analysis of Transcriptomics Data within Network Biomedicine Perspective. PLoS One 13, e0200717. 10.1371/journal.pone.0200717 30020984 PMC6051662

[B78] Kortel M. Mansuriya B. D. Vargas Santana N. Altintas Z. (2020). Graphene Quantum Dots as Flourishing Nanomaterials for Bio-Imaging, Therapy Development, and Micro-supercapacitors. Micromachines (Basel) 11. 866. 10.3390/mi11090866 PMC7570118 32962061

[B79] Kumar Y. R. Deshmukh K. Sadasivuni K. K. Pasha S. K. K. (2020). Graphene Quantum Dot Based Materials for Sensing, Bio-Imaging and Energy Storage Applications: a Review. RSC Adv. 10, 23861–23898. 10.1039/d0ra03938a PMC9055121 35517370

[B80] Lai H. Stenzel M. H. Xiao P. (2020). Surface Engineering and Applications of Nanodiamonds in Cancer Treatment and Imaging. Int. Mater. Rev. 65, 189–225. 10.1080/09506608.2019.1622202

[B81] Li J.-J. Shang L. Jia L.-P. Ma R.-N. Zhang W. Jia W.-L. (2018). An Ultrasensitive Electrochemiluminescence Sensor for the Detection of HULC Based on Au@Ag/GQDs as a Signal Indicator. J. Electroanalytical Chem. 824, 114–120. 10.1016/j.jelechem.2018.07.044

[B82] Liguori C. Frauenfelder G. Massaroni C. Saccomandi P. Giurazza F. Pitocco F. (2015). Emerging Clinical Applications of Computed Tomography. Med. Devices (Auckl) 8, 265–278. 10.2147/MDER.S70630 26089707 PMC4467659

[B83] Lim S. Y. Shen W. Gao Z. (2015). Carbon Quantum Dots and Their Applications. Chem. Soc. Rev. 44, 362–381. 10.1039/c4cs00269e 25316556

[B84] Liu K. Zhao T. Wang J. Chen Y. Zhang R. Lan X. (2019). Etiology, Cancer Stem Cells and Potential Diagnostic Biomarkers for Esophageal Cancer. Cancer Lett. 458, 21–28. 10.1016/j.canlet.2019.05.018 31125642 PMC6597177

[B85] Liu Q. Xu S. Niu C. Li M. He D. Lu Z. (2015). Distinguish Cancer Cells Based on Targeting Turn-On Fluorescence Imaging by Folate Functionalized green Emitting Carbon Dots. Biosens. Bioelectron. 64, 119–125. 10.1016/j.bios.2014.08.052 25203943

[B86] Liu W. Wei J. Chen Y. (2014). Electrospun Poly(l-Lactide) Nanofibers Loaded with Paclitaxel and Water-Soluble Fullerenes for Drug Delivery and Bioimaging. New J. Chem. 38, 6223–6229. 10.1039/c4nj01259c

[B87] Liu Y.-Y. Chang B.-M. Chang H.-C. (2020). Nanodiamond-enabled Biomedical Imaging. Nanomedicine 15, 1599–1616. 10.2217/nnm-2020-0091 32662335

[B88] Liu Y. Li L. Yu J. Fan Y.-X. Lu X.-B. (2018). Carbon Nanoparticle Lymph Node Tracer Improves the Outcomes of Surgical Treatment in Papillary Thyroid Cancer. Cancer. Biomark. 23, 227–233. 10.3233/cbm-181386 30198867 PMC13078537

[B89] Lizzi F. L. Feleppa E. J. Kaisar Alam S. Deng C. X. (2003). Ultrasonic Spectrum Analysis for Tissue Evaluation. Pattern Recognition Lett. 24, 637–658. 10.1016/s0167-8655(02)00172-1

[B90] Lu G.-H. Shang W.-T. Deng H. Han Z.-Y. Hu M. Liang X.-Y. (2019). Targeting Carbon Nanotubes Based on IGF-1R for Photothermal Therapy of Orthotopic Pancreatic Cancer Guided by Optical Imaging. Biomaterials 195, 13–22. 10.1016/j.biomaterials.2018.12.025 30599289

[B91] Mahajan S. Patharkar A. Kuche K. Maheshwari R. Deb P. K. Kalia K. (2018). Functionalized Carbon Nanotubes as Emerging Delivery System for the Treatment of Cancer. Int. J. Pharmaceutics 548, 540–558. 10.1016/j.ijpharm.2018.07.027 29997043

[B92] Maleki R. Khoshoei A. Ghasemy E. Rashidi A. (2020). Molecular Insight into the Smart Functionalized TMC-Fullerene Nanocarrier in the pH-Responsive Adsorption and Release of Anti-cancer Drugs. J. Mol. Graphics Model. 100, 107660. 10.1016/j.jmgm.2020.107660 32659627

[B93] Martín-Gracia B. Martín-Barreiro A. Cuestas-Ayllón C. Grazú V. Line A. Llorente A. (2020). Nanoparticle-based Biosensors for Detection of Extracellular Vesicles in Liquid Biopsies. J. Mater. Chem. B 8, 6710–6738. 10.1039/d0tb00861c 32627783

[B94] Martínez-Jothar L. Doulkeridou S. Schiffelers R. M. Sastre Torano J. Oliveira S. Van Nostrum C. F. (2018). Insights into Maleimide-Thiol Conjugation Chemistry: Conditions for Efficient Surface Functionalization of Nanoparticles for Receptor Targeting. J. Controlled Release 282, 101–109. 10.1016/j.jconrel.2018.03.002 29526739

[B95] Matshitse R. Tshiwawa T. Managa M. Nwaji N. Lobb K. Nyokong T. (2020). Theoretical and Photodynamic Therapy Characteristics of Heteroatom Doped Detonation Nanodiamonds Linked to Asymmetrical Phthalocyanine for Eradication of Breast Cancer Cells. J. Lumin. 227, 117465. 10.1016/j.jlumin.2020.117465

[B96] Matsuoka T. Yashiro M. (2018). Biomarkers of Gastric Cancer: Current Topics and Future Perspective. Wjg 24, 2818–2832. 10.3748/wjg.v24.i26.2818 30018477 PMC6048430

[B98] Nasrollahi F. Koh Y. R. Chen P. Varshosaz J. Khodadadi A. A. Lim S. (2019). Targeting Graphene Quantum Dots to Epidermal Growth Factor Receptor for Delivery of Cisplatin and Cellular Imaging. Mater. Sci. Eng. C 94, 247–257. 10.1016/j.msec.2018.09.020 30423706

[B99] Nitta H. Minami K. Harano K. Nakamura E. (2015). DNA Binding of Pentaamino[60]fullerene Synthesized Using Click Chemistry. Chem. Lett. 44, 378–380. 10.1246/cl.141092

[B100] Othman H. O. Salehnia F. Hosseini M. Hassan R. Faizullah A. Ganjali M. R. (2020). Fluorescence Immunoassay Based on Nitrogen Doped Carbon Dots for the Detection of Human Nuclear Matrix Protein NMP22 as Biomarker for Early Stage Diagnosis of Bladder Cancer. Microchemical J. 157, 104966. 10.1016/j.microc.2020.104966

[B101] Pardo J. Peng Z. Leblanc R. M. (2018). Cancer Targeting and Drug Delivery Using Carbon-Based Quantum Dots and Nanotubes. Molecules 23. 10.3390/molecules23020378 PMC6017112 29439409

[B102] Parikh N. D. Mehta A. S. Singal A. G. Block T. Marrero J. A. Lok A. S. (2020). Biomarkers for the Early Detection of Hepatocellular Carcinoma. Cancer Epidemiol. Biomarkers Prev. 29, 2495–2503. 10.1158/1055-9965.EPI-20-0005 32238405 PMC7529652

[B103] Perevedentseva E. Lin Y. C. Cheng C. L. (2020). A Review of Recent Advances in Nanodiamond-Mediated Drug Delivery in Cancer. Expert Opin. Drug Deliv., 1–14. 10.1080/17425247.2021.1832988 33047984

[B104] Pugash D. Brugger P. C. Bettelheim D. Prayer D. (2008). Prenatal Ultrasound and Fetal MRI: the Comparative Value of Each Modality in Prenatal Diagnosis. Eur. J. Radiol. 68, 214–226. 10.1016/j.ejrad.2008.06.031 18790583

[B105] Qin S.-R. Zhao Q. Cheng Z.-G. Zhang D.-X. Zhang K.-K. Su L.-X. (2019). Rare Earth-Functionalized Nanodiamonds for Dual-Modal Imaging and Drug Delivery. Diamond Relat. Mater. 91, 173–182. 10.1016/j.diamond.2018.11.015

[B106] Quintero-Jaime A. F. Berenguer-Murcia Á. Cazorla-Amorós D. Morallón E. (2019). Carbon Nanotubes Modified with Au for Electrochemical Detection of Prostate Specific Antigen: Effect of Au Nanoparticle Size Distribution. Front. Chem. 7. 147. 10.3389/fchem.2019.00147 30972319 PMC6445958

[B107] Ravi Kiran A. V. V. V. Kusuma Kumari G. Krishnamurthy P. T. (2020). Carbon Nanotubes in Drug Delivery: Focus on Anticancer Therapies. J. Drug Deliv. Sci. Tech. 59, 101892. 10.1016/j.jddst.2020.101892

[B108] Reineck P. Abraham A. N. Poddar A. Shukla R. Abe H. Ohshima T. (2021). Multimodal Imaging and Soft X-Ray Tomography of Fluorescent Nanodiamonds in Cancer Cells. Biotechnol. J. 16, e2000289. 10.1002/biot.202000289 32975037

[B109] Rezaian M. Maleki R. Dahri Dahroud M. Alamdari A. Alimohammadi M. (2018). pH-Sensitive Co-Adsorption/Release of Doxorubicin and Paclitaxel by Carbon Nanotube, Fullerene, and Graphene Oxide in Combination with N-Isopropylacrylamide: A Molecular Dynamics Study. Biomolecules 8. 127. 10.3390/biom8040127 PMC6316683 30380660

[B110] Saallah S. Lenggoro I. W. (2018). Nanoparticles Carrying Biological Molecules: Recent Advances and Applications. Kona 35, 89–111. 10.14356/kona.2018015

[B111] Sanginario A. Miccoli B. Demarchi D. (2017). Carbon Nanotubes as an Effective Opportunity for Cancer Diagnosis and Treatment. Biosensors (Basel) 7. 9. 10.3390/bios7010009 PMC5371782 28212271

[B112] Sapsford K. E. Algar W. R. Berti L. Gemmill K. B. Casey B. J. Oh E. (2013). Functionalizing Nanoparticles with Biological Molecules: Developing Chemistries that Facilitate Nanotechnology. Chem. Rev. 113, 1904–2074. 10.1021/cr300143v 23432378

[B113] Sergeeva V. Kraevaya O. Ershova E. Kameneva L. Malinovskaya E. Dolgikh O. (2019). Antioxidant Properties of Fullerene Derivatives Depend on Their Chemical Structure: A Study of Two Fullerene Derivatives on HELFs. Oxid Med. Cel Longev 2019, 4398695. 10.1155/2019/4398695 PMC6360044 30800207

[B145] Sharma H. Mondal S. (2020). Functionalized Graphene Oxide for Chemotherapeutic Drug Delivery and Cancer Treatment: A Promising Material in Nanomedicine. Int. J. Mol. Sci. 21(17), 6280. 10.3390/ijms21176280 PMC7504176 32872646

[B114] Shi J. Wang L. Gao J. Liu Y. Zhang J. Ma R. (2014). A Fullerene-Based Multi-Functional Nanoplatform for Cancer Theranostic Applications. Biomaterials 35, 5771–5784. 10.1016/j.biomaterials.2014.03.071 24746227

[B115] Skivka L. M. Prylutska S. V. Rudyk M. P. Khranovska N. M. Opeida I. V. Hurmach V. V. (2018). C(60) Fullerene and its Nanocomplexes with Anticancer Drugs Modulate Circulating Phagocyte Functions and Dramatically Increase ROS Generation in Transformed Monocytes. Cancer Nanotechnol 9, 8. 10.1186/s12645-017-0034-0 30416604 PMC6208740

[B116] Song Y. Shi W. Chen W. Li X. Ma H. (2012). Fluorescent Carbon Nanodots Conjugated with Folic Acid for Distinguishing Folate-Receptor-Positive Cancer Cells from normal Cells. J. Mater. Chem. 22, 12568–12573. 10.1039/c2jm31582c

[B117] Su W. Guo R. Yuan F. Li Y. Li X. Zhang Y. (2020). Red-Emissive Carbon Quantum Dots for Nuclear Drug Delivery in Cancer Stem Cells. J. Phys. Chem. Lett. 11, 1357–1363. 10.1021/acs.jpclett.9b03891 32017568

[B118] Sung H. Ferlay J. Siegel R. L. Laversanne M. Soerjomataram I. Jemal A. (2021). Global Cancer Statistics 2020: GLOBOCAN Estimates of Incidence and Mortality Worldwide for 36 Cancers in 185 Countries. CA Cancer J. Clin. 79, 209–249. 10.3322/caac.21660 33538338

[B119] Tiron C. E. Luta G. Butura M. Zugun-Eloae F. Stan C. S. Coroaba A. (2020). NHF-derived Carbon Dots: Prevalidation Approach in Breast Cancer Treatment. Scientific Rep. 10, 12662. 10.1038/s41598-020-69670-z PMC7391642 32728167

[B120] Vacante M. Borzì A. M. Basile F. Biondi A. (2018). Biomarkers in Colorectal Cancer: Current Clinical Utility and Future Perspectives. Would. J. Clin. Case. 6, 869–881. 10.12998/wjcc.v6.i15.869 PMC6288499 30568941

[B121] Valcourt D. M. Harris J. Riley R. S. Dang M. Wang J. Day E. S. (2018). Advances in Targeted Nanotherapeutics: From Bioconjugation to Biomimicry. Nano Res. 11, 4999–5016. 10.1007/s12274-018-2083-z 31772723 PMC6879063

[B122] Villalobos P. Wistuba I. I. (2017). Lung Cancer Biomarkers. Hematology/Oncology Clin. North America 31, 13–29. 10.1016/j.hoc.2016.08.006 PMC5137804 27912828

[B123] Wang J. Wang L. Zhu Y. Zhang J. Liao J. Wang S. (2016). A High Accuracy Cantilever Array Sensor for Early Liver Cancer Diagnosis. Biomed. Microdevices 18, 110. 10.1007/s10544-016-0132-5 27834053

[B124] Wang X. Qian X. Beitler J. J. Chen Z. G. Khuri F. R. Lewis M. M. (2011). Detection of Circulating Tumor Cells in Human Peripheral Blood Using Surface-Enhanced Raman Scattering Nanoparticles. Cancer Res. 71, 1526–1532. 10.1158/0008-5472.can-10-3069 21212408 PMC3079317

[B125] Wang Y. Hu A. (2014). Carbon Quantum Dots: Synthesis, Properties and Applications. J. Mater. Chem. C 2, 6921–6939. 10.1039/c4tc00988f

[B126] Wei S. Li L. Du X. Li Y. (2019). OFF-ON Nanodiamond Drug Platform for Targeted Cancer Imaging and Therapy. J. Mater. Chem. B 7, 3390–3402. 10.1039/c9tb00447e

[B127] Williams R. M. Lee C. Heller D. A. (2018). A Fluorescent Carbon Nanotube Sensor Detects the Metastatic Prostate Cancer Biomarker uPA. ACS Sens. 3, 1838–1845. 10.1021/acssensors.8b00631 30169018 PMC11929042

[B128] Xiang C. Zhang Y. Guo W. Liang X.-J. (2020). Biomimetic Carbon Nanotubes for Neurological Disease Therapeutics as Inherent Medication. Acta Pharmaceutica Sinica B 10, 239–248. 10.1016/j.apsb.2019.11.003 32082970 PMC7016289

[B129] Xiao Q. Jia Q. Tan J. Meng Z. (2020). Serum Biomarkers for Thyroid Cancer. Biomarkers Med. 14, 807–815. 10.2217/bmm-2019-0578 32677454

[B130] Xu Y. Li P. Cheng D. Wu C. Lu Q. Yang W. (2020). Group IV Nanodots: Synthesis, Surface Engineering and Application in Bioimaging and Biotherapy. J. Mater. Chem. B 8, 10290–10308. 10.1039/d0tb01881c 33103712

[B131] Yan J. Zheng X. Liu Z. Yu J. Deng Z. Xue F. (2016). A Multicenter Study of Using Carbon Nanoparticles to Show sentinel Lymph Nodes in Early Gastric Cancer. Surg. Endosc. 30, 1294–1300. 10.1007/s00464-015-4358-8 26150223

[B132] Ye F. Zhao Y. El-Sayed R. Muhammed M. Hassan M. (2018). Advances in Nanotechnology for Cancer Biomarkers. Nano Today 18, 103–123. 10.1016/j.nantod.2017.12.008

[B133] Younis M. R. He G. Lin J. Huang P. (2020). Recent Advances on Graphene Quantum Dots for Bioimaging Applications. Front. Chem. 8, 424. 10.3389/fchem.2020.00424 32582629 PMC7283876

[B134] Yu B. Tan L. Zheng R. Tan H. Zheng L. (2016). Targeted Delivery and Controlled Release of Paclitaxel for the Treatment of Lung Cancer Using Single-Walled Carbon Nanotubes. Mater. Sci. Eng. C 68, 579–584. 10.1016/j.msec.2016.06.025 27524057

[B135] Yu Y. Yang X. Liu M. Nishikawa M. Tei T. Miyako E. (2019). Multifunctional Cancer Phototherapy Using Fluorophore-Functionalized Nanodiamond Supraparticles. ACS Appl. Bio Mater. 2, 3693–3705. 10.1021/acsabm.9b00603 35030756

[B136] Yuan Y.-G. Gurunathan S. (2017). Combination of Graphene Oxide–Silver Nanoparticle Nanocomposites and Cisplatin Enhances Apoptosis and Autophagy in Human Cervical Cancer Cells. Int. J. nanomedicine. Vol. 12, 6537–6558. 10.2147/ijn.s125281 PMC5592952 28919753

[B137] Zhang J.-J. Cao J.-T. Shi G.-F. Liu Y.-M. Chen Y.-H. Ren S.-W. (2015). Sandwich-format Electrochemiluminescence Assay for PDGF-BB Using Quantum Dots-Dendrimer Nanocomposites as Probe. Talanta 141, 158–163. 10.1016/j.talanta.2015.04.001 25966396

[B138] Zhang J. Liu Z. Zhou S. Teng Y. Zhang X. Li J. (2020a). Novel Span-PEG Multifunctional Ultrasound Contrast Agent Based on CNTs as a Magnetic Targeting Factor and a Drug Carrier. ACS Omega 5, 31525–31534. 10.1021/acsomega.0c03325 33344804 PMC7745219

[B139] Zhang J. Song L. Zhou S. Hu M. Jiao Y. Teng Y. (2019). Enhanced Ultrasound Imaging and Anti-tumor *In Vivo* Properties of Span-Polyethylene Glycol with Folic Acid-Carbon Nanotube-Paclitaxel Multifunctional Microbubbles. RSC Adv. 9, 35345–35355. 10.1039/c9ra06437k PMC9074749 35528086

[B140] Zhang L. Yao M. Yan W. Liu X. Jiang B. Qian Z. (2017). Delivery of a Chemotherapeutic Drug Using Novel Hollow Carbon Spheres for Esophageal Cancer Treatment. Int. J. Nanomedicine. Vol. 12, 6759–6769. 10.2147/ijn.s142916 PMC5600264 28932119

[B141] Zhang Q. Deng S. Liu J. Zhong X. He J. Chen X. (2018). Cancer‐Targeting Graphene Quantum Dots: Fluorescence Quantum Yields, Stability, and Cell Selectivity. Adv. Funct. Mater. 29, 1805860. 10.1002/adfm.201805860

[B142] Zhang Y. Li S. Ma X.-T. He X.-W. Li W.-Y. Zhang Y.-K. (2020b). Carbon Dots-Embedded Epitope Imprinted Polymer for Targeted Fluorescence Imaging of Cervical Cancer via Recognition of Epidermal Growth Factor Receptor. Microchimica Acta 187, 228. 10.1007/s00604-020-4198-7 32170469

[B143] Zhao C. Song X. Liu Y. Fu Y. Ye L. Wang N. (2020). Synthesis of Graphene Quantum Dots and Their Applications in Drug Delivery. J. Nanobiotechnology 18, 142. 10.1186/s12951-020-00698-z 33008457 PMC7532648

[B144] Zhou W. Gao X. Liu D. Chen X. (2015). Gold Nanoparticles for *In Vitro* Diagnostics. Chem. Rev. 115, 10575–10636. 10.1021/acs.chemrev.5b00100 26114396 PMC5226399

