# Supplementary material for: Carbon Based Nanodots in Early Diagnosis of Cancer
Source: Front Chem. 2021 May 24;9:669169. doi: 10.3389/fchem.2021.669169 (PMC8181141; doi:10.3389/fchem.2021.669169)
Supplement: Supplementary file 1 [file DataSheet1.doc]

**Supplementary information**

**Extended introduction**

There is an increased interest in carbon-based nanomaterials for disease diagnosis and treatment. Efficient nanomaterials that can more readily target cancer cells will improve the prevention strategies and treatment outcomes. This could be accomplished by the combined endeavors of nanotoxicologists, cancer biologists and nanobiomaterial researchers being focused on cancer treatment. Designing carbon-based nanomaterials is among the most sought-after fields in modern advanced materials science and engineering. Till now, different carbon nanomaterials, for example, carbon quantum dots (CQDs), graphene quantum dots (GQDs), nanodiamonds, fullerenes (C_60_), carbon nanotubes (CNTs) and nanocantilevers have been extensively researched upon. These are being utilized in bioimaging, drug delivery, nucleus targeting and labeling, photodynamic therapy (PDT), photocatalyst design, photodetectors, and numerous other biological and engineering fields. Among these nanotechnology tools, carbon based nanodots have garnered attention because of their smaller size (< 10 nm), robust chemical inertness, good photostability, high quantum yield etc. Through easy, inexpensive and well-developed synthetic approaches, carbon based nanodots can be effectively delivered from different common resources like glucose, wool, acid and insecticide. These distinctive attributes make carbon based nanodots successful competitors of fluorescent nanoprobes for various purposes such as bioimaging, drug delivery and transgene therapy.

During the past few years, CQDs have progressively become important because of their, abundance and low-cost. CQDs have received increased recognition due to their properties like great biocompatibility, low biotoxicity, chemical stability and the possibility of functionalization; consequently these are potential tools for bioimaging, drug delivery, gene delivery and cancer therapy. The spearheading work on CQDs for bioimaging (*in vitro* and *in vivo*) was reported by Sun's group (Sun et al., 2020). Here, confocal microscopy pictures of *Escherichia* *coli* ATCC 25922 tagged with the PEGylated CQDs were acquired at various excitation wavelengths. CQDs have been utilized as biosensor carriers for their high solubility in water, flexibility in surface modifications, non-toxicity, multiplexing, incredible biocompatibility, and high photostability. Another example of the most recent members from carbon based nanomaterials family is GQDs. Ponomarenko and Geim are accredited with the highlighting the importance of GQDs (Ponomarenko et al., 2008). The enormous interest in GQDs emerges from the fact that these are composed of carbon, quite possibly the most plentiful component on earth, and can possibly substitute semiconducting quantum dots (QDs) in certain applications. Also, the carbon based arrangement of GQDs render them low toxicity, compatibility to biological systems, insignificant environmental impact and better photostability, facilitating their utilization in biomedical and biological applications. In correlation with semiconducting QDs, GQDs exhibit stability against blinking and photobleaching, which is an essential feature for its biological applications. Notwithstanding, the literature has revealed that tumor microenvironment modulates nanostructures physicochemically (Sun et al., 2017;Cao et al., 2019;Li et al., 2020). The size, shape, and surface functionalities of GQDs are accountable for their cellular responses, *viz.* uptake, accrual, and cytotoxicity. It was noticed that high aqueous dispersibility, large surface/volume ratio and surface functionalities make GQDs ideal candidate for custom adjustments for specific targets and cellular models. Additionally, GQDs can be efficiently modulated to display ideal results in cancer treatment like tumor accumulation, drug release, and numerous imaging options. Other than verifiable outcomes, there is a need to examine the obstacles for use of GQDs in theranostic applications in clinical use. In general, *in vitro* as well as *in vivo* studies predict a decent future for GQDs as theranostics, which will be of utmost significance in cancer treatment (Tian et al., 2018).

Nanodiamonds are a novel class of carbon material, which shows high compatibility with biological systems, and have other beneficial qualities previously mentioned. Close to their astounding biocompatibility, they are very flexible to bind and deliver various drugs, imaging agents, nucleic acids and proteins. Few investigations depict that an additional coating of nanodiamonds with a targeting agent or cell penetrating peptide lead to a more compelling anti-cancer effect. The first proof of the use of nanodiamonds in cancer treatment was published in 2010 (Guan et al., 2010). It has been reported that nanodiamonds considerably reduced the proliferation of human cervical cells by delivery of cisplatin and its release in an acidic intracellular surroundings. An outstanding investigation reported the utilization of nanodiamonds for the treatment of glioblastoma. Here, nanodiamonds tend to serve as a delivery vehicle for doxorubicin (DOX) through a convection-enhanced delivery (CED). The CED approach is believed to overcome the lack of penetration in blood brain barrier. This barrier signifies the fundamental obstacle in the therapy of glioblastoma, since it forestalls the adequate accumulation of anticancer drugs in the brain after systemic administration (Xi et al., 2014).

C_60_ is an exceptional type of carbon based nanomaterial with molecules comprising of 60 carbon atoms, which appear like soccer ball. In order to make C_60_ soluble in water and biological solutions, polar functional groups are chemically appended to their surfaces. Initially, C_60_ and its derivatives have been considered as the most encouraging nanomaterials on account of their extraordinary properties for medicinal applications. These are capable of delivering drugs or small therapeutic molecules to cancer cells. Of late, a group of researchers have discovered that some of the C_60_ compounds are toxic for non-small cell lung carcinoma cells and cause their destruction (Huang et al., 2021). Simultaneously, they are basically non-toxic for healthy cells. Henceforth, the anti-tumor activity of the C_60_ derivatives has an immense potential for the development of novel compelling restorative products for treatment of non-small cell lung carcinoma. C_60_ and its derivatives have been affirmed for their potential as drug vehicles in cancer chemotherapy. Moreover, C_60_ and its derivatives were additionally used to inhibit growth of bacterial pathogens like *Escherichia* *coli*, *Shewanella* *oneidensis* etc. (Al-Jumaili et al., 2017).

Lately, carbon nanotubes (CNTs) have emerged as novel carriers for therapeutics varying in size, ranging from small to large in size. Their biological and physical properties can be easily modulated by functionalization. This feature is particularly important for targeting cancer cells by means of photothermal therapy (PTT).

Cancer cells are known to express folic acid (FA) receptors; this property has been exploited to synthesize different nanocarriers that can attach these FA derivatives.(Feng et al., 2013;Zhang et al., 2018b). Additionally, non-spherical nanocarriers CNTs were held in lymph nodes for considerable time in comparison to spherical nanocarriers (e.g., liposomes). In this way, CNTs may be utilized for targeting lymphatic cancers as demonstrated by another group (Falank et al., 2019). In another study, a chemotherapeutic alkaloidcamptothecin was loaded into multivalent CNTs and it was observed to be an effective approach for breast and skin cancer therapies (Sahoo et al., 2011).

Lastly, designed nanocantilevers show significantly low toxicity and high biocompatibility. The advantages associated with nanocantilevers include their usage for cancer detection without any fluorescent or radioactive labeling agents. The detection may be done in liquid samples and this know-how might be effectively translated to lab-on-a-chip techniques, rendering point of-care diagnostics. Nanoscale cantilever tools, in which cantilevers are covered with explicit receptors, may give profoundly sensitive and rapid detection of disease specific molecules, like DNA or protein. Albeit a few problems and hindrances are hampering the development of nano tools, the capability of nanotechnologies to function as multimodal nanotheranostic tool will probably make these tools ready for battle against different cancer types and encourage the progress of customized treatment for early diagnosis and treatment of cancer.

**Importance of nanotechnology in developing early diagnostic tools**

Diagnosis and treatment of cancer is very tedious and time-consuming. The common treatments include multiple surgeries, testing for biomarkers, immunotherapy, chemotherapy and radiation therapy, all of which have certain pros and cons. Here, delivery of chemotherapeutics and subsequent diagnosis are dependent on novel interventions based on nanotechnology. Due to high selectivity and sensitivity offered by nanotechnology based tools, numerous targets can be analyzed simultaneously (Zhang et al., 2019). Their favorable attributes such as surface chemistry, precise architecture, etc. provide distinct biological properties, making them suitable to deliver different molecules (Thakor and Gambhir, 2013). Nanotechnology has been effectively used to detect multiple cancer biomarkers, since detection of these biomarkers is associated with early diagnosis of cancer and helps to keep a check on effectiveness of the therapy (Zhang et al., 2019). Detection of circulating tumor cells (CTCs), associated with metastasis of cancer has been effected with the help of nanotechnology, as evidenced by recent studies (Liu et al., 2014;Huang et al., 2018). NPs accumulate in tumor tissues and thus, allow imaging of the tumor and its diagnosis *in vivo*. The delivery of these NPs can be carried out via active or passive targeting (Zhang et al., 2019).

Carbon based nanodots have shown promising outcomes as of late. These nanodots permit diagnosis and delivery of chemotherapeutics, which involves photophysical and photochemical responses triggered by light. Further, these can be utilized in malignancy treatment, photodynamic treatment and photothermal treatment (Adrita et al., 2020;Qin et al., 2021). Additionally, the accessible –COOH group on carbon based nanoparticles permits zero-length carboxyl-to-amine crosslinking to take place. In such manner, protein-based therapeutics, small molecule drugs and targeting ligands with free NH_2_ group are best suited for conjugation. Accordingly, carbon-based nanoparticles have been demonstrated to be powerful for drug delivery and tumor diagnosis. Carbon particles are nontoxic, unlike a few other heavier, metal-based nanoparticles, and possess functional groups similar in number and quantity to that of polymers like polyethylene glycol (PEG) regularly used in the field. Despite the fact that carbon nanodots-based cancer therapeutics are at initial stages, they have the specialized ability to build up a perfect drug delivery and detection system that can bring new hope for diagnosing, treating, and forestalling malignant growth.

**Cancer biomarkers**

Biomarkers have enormous prospective in tumor biology for malignancy screening, diagnosis, prognosis and remedial targets. These biomarkers could be DNA-based, RNA-based, protein-based, and antibody based (Jayanthi et al., 2017). In malignancy research and detection, a biomarker simply refers to a molecule that is demonstrative of the presence of cancer in the body. These can be detected in tissues, cells, and fluids. To amplify the worth and limit the expense for screening, it is beneficial for these biomarkers to be quantifiable in body fluids like serum, urine, and sputum.

Some typical proteins are generally overexpressed in cancer cells, which can be used for its early detection. Clinically, a few crucial tumor markers such as prostate specific antigen (PSA), alpha-fetoprotein (AFP), carcinoembryonic antigen (CEA), carbohydrate antigen 19-9 (CA19-9), etc. have been utilized in the diagnosis of certain cancers (Bhatt et al., 2010). But, low sensitivity of these biomarkers for early cancer detection limits their applicability and so, these are currently just being used in choosing prognosis, foreseeing treatment responses, and management of surgical treatment. Quite possibly, the main role of cancer biomarkers in coming times would be to use them in inescapable screening, so that asymptomatic individuals can also be screened for disease at an early stage. Shockingly, there are no molecular cancer markers that can be perceived as successful for evaluating huge segments of the populace for the presence of at least one cancer type. So far, the only tumor biomarker approved by US Food and Drug Administration (FDA) for inescapable screening designs is PSA, alongside digital rectal examination (DRE). In spite of the achievement of PSA in detecting early stage prostate cancer in certain individuals, its utilization to evaluate patients for prostate cancer stays disputable. Besides, it is not clear whether the advantages of the PSA test exceed the possibility of follow-up diagnostic tests and treatment (Liu et al., 2019).

Among all cancer types, bladder cancer is the sixth most common disease among men as well as women. A majority of patients with recently diagnosed bladder malignancy have shallow, substandard neoplasms that are related with an inconceivable prognosis. These tumors have a 30% to 70% repeat rate and may advance to intrusive diseases in 10% to 30% of patients. Hence, the early detection of bladder tumors is fundamental but important for improved patient diagnosis and to improve survival rates. Cytology and cystoscopy have been utilized as detection tests for patients suspected for bladder malignancy. Cystoscopy is exceptionally sensitive for most tumors; however it has limitations of being obtrusive and shows poor sensitivity. Accordingly, non-obtrusive biomarkers (BCLA-1, BCLA-4, NMP-22, BTA Stat and BTA-TRAK) have been searched for as alternatives to cystoscopy and cytology for investigating bladder malignancy (Santoni et al., 2018). Among females, breast malignancy is the most commonly diagnosed disease, with 2.1 million cases diagnosed in 2018, representing practically 25% malignancy cases among women. Breast malignancy is also the main source of malignancy death in more than 100 nations, adding to 15% of all cancer death in 2018. The prognosis is exceptionally poor when cancer is diagnosed at an advanced stage. Many screening strategies have been utilized for early detection of breast cancer; however there are few limitations of these techniques. Several efforts have been made to investigate biomarkers (Estrogen receptor (ER), progesterone receptor (PR), atomic antigen Ki-67, and human epidermal development factor receptor (HER-2)) for early detection of breast malignancy (Dai et al., 2016;Duffy et al., 2017;Nicolini et al., 2018). Additionally, for different cancers (cervical, colorectal, liver, lung, stomach, thyroid and so forth) many biomarkers have been investigated to conquer the disarray; the quest for more sensitive and specific cancer biomarkers is ongoing. The eventual fate of malignancy management is dependent upon the use of biomarkers that will direct physicians at each step of cancer management.

**Current challenges**

Even though carbon based nanomaterials have an edge over conventional diagnostic techniques for early detection as well as treatment of cancer, these nanotechnology-based tools suffer from certain shortcomings that limit their wide scale applicability to biological systems. The *in vivo* effects of the nanomaterials need to be carefully evaluated for their toxicity to normal cells in vicinity to cancer cells. Pharmacological aspects as well as toxicology-based studies need to be taken into consideration before these nanomaterials are approved for large-scale usage. Many *in vivo* efficacy studies are needed in order for these carbon based nanomaterials to qualify as safe for diagnosis of cancer and for wider applicability (Bianco et al., 2008). For instance, carbon nanotubes despite their applications in many fields are considerably toxic to biological systems as revealed by many studies (Cimbaluk et al., 2018;Zhang et al., 2018a;Zhao et al., 2021). Impurities, like presence of metal ions, added during synthesis of CNTs majorly impact their toxicity. Retention of CNTs for a long time has been found to cause inflammation, ultimately leading to progressive fibrosis (Maiti et al., 2019). Similarly, toxicity of graphene derivatives to biological systems is also proven (Dasari Shareena et al., 2018). Further, the use of CDs is arguable due to their *in vivo* toxicity, synthetic materials being utilized for synthesisfunctional groups, and wide array of size distribution. Factors like toxicity, , rapid elimination from the body and hydrodynamic diameter should be considered while designing CDs.

Nevertheless, because of their fascinating properties, carbon nanomaterials have been proposed as good tools in identifying distinctive biological molecules at early stages of cancer. Additionally, their usage can save time in comparison to the traditional ELISA commercial test kits. Moreover, this methodology is promising for developing handheld tools at low cost compared with techniques currently in use, like immunoassays, test-strips, and kits. In brief, carbon based nanomaterial will start being extensively used for clinical investigations like diagnosis and treatment of cancer in near future.

***Supplementary Figure 1.*** *Bioconjugation of nanoparticles with different functional groups (****A)*** *A schematic of carboxylic group coupling with folic acid, antibodies and peptide* ***(B)*** *Succinimidyl 4-(N-maleimidomethyl)cyclohexane-1-carboxylate (SMCC) activated amine functionalized nanoparticle coupled to thiol functionalized protein moiety.*

**References**

Adrita, S.H., Tasnim, K.N., Ryu, J.H., and Sharker, S.M. (2020). Nanotheranostic Carbon Dots as an Emerging Platform for Cancer Therapy. *Journal of Nanotheranostics* 1**,** 58-77.

Al-Jumaili, A., Alancherry, S., Bazaka, K., and Jacob, M.V. (2017). Review on the Antimicrobial Properties of Carbon Nanostructures. *Materials (Basel, Switzerland)* 10**,** 1066.

Bhatt, A.N., Mathur, R., Farooque, A., Verma, A., and Dwarakanath, B.S. (2010). Cancer biomarkers - current perspectives. *Indian J Med Res* 132**,** 129-149.

Bianco, A., Kostarelos, K., and Prato, M. (2008). Opportunities and challenges of carbon-based nanomaterials for cancer therapy. *Expert Opin Drug Deliv* 5**,** 331-342.

Cao, M., Lu, S., Wang, N., Xu, H., Cox, H., Li, R., Waigh, T., Han, Y., Wang, Y., and Lu, J.R. (2019). Enzyme-Triggered Morphological Transition of Peptide Nanostructures for Tumor-Targeted Drug Delivery and Enhanced Cancer Therapy. *ACS Applied Materials & Interfaces* 11**,** 16357-16366.

Cimbaluk, G.V., Ramsdorf, W.A., Perussolo, M.C., Santos, H.K.F., Da Silva De Assis, H.C., Schnitzler, M.C., Schnitzler, D.C., Carneiro, P.G., and Cestari, M.M. (2018). Evaluation of multiwalled carbon nanotubes toxicity in two fish species. *Ecotoxicology and Environmental Safety* 150**,** 215-223.

Dai, X., Xiang, L., Li, T., and Bai, Z. (2016). Cancer Hallmarks, Biomarkers and Breast Cancer Molecular Subtypes. *Journal of Cancer* 7**,** 1281-1294.

Dasari Shareena, T.P., Mcshan, D., Dasmahapatra, A.K., and Tchounwou, P.B. (2018). A Review on Graphene-Based Nanomaterials in Biomedical Applications and Risks in Environment and Health. *Nano-Micro Letters* 10**,** 53.

Duffy, M.J., Harbeck, N., Nap, M., Molina, R., Nicolini, A., Senkus, E., and Cardoso, F. (2017). Clinical use of biomarkers in breast cancer: Updated guidelines from the European Group on Tumor Markers (EGTM). *European Journal of Cancer* 75**,** 284-298.

Falank, C., Tasset, A.W., Farrell, M., Harris, S., Everill, P., Marinkovic, M., and Reagan, M.R. (2019). Development of medical-grade, discrete, multi-walled carbon nanotubes as drug delivery molecules to enhance the treatment of hematological malignancies. *Nanomedicine: Nanotechnology, Biology and Medicine* 20**,** 102025.

Feng, D., Song, Y., Shi, W., Li, X., and Ma, H. (2013). Distinguishing folate-receptor-positive cells from folate-receptor-negative cells using a fluorescence off-on nanoprobe. *Anal Chem* 85**,** 6530-6535.

Guan, B., Zou, F., and Zhi, J. (2010). Nanodiamond as the pH-Responsive Vehicle for an Anticancer Drug. *Small* 6**,** 1514-1519.

Huang, H.-J., Chetyrkina, M., Wong, C.-W., Kraevaya, O.A., Zhilenkov, A.V., Voronov, I.I., Wang, P.-H., Troshin, P.A., and Hsu, S.-H. (2021). Identification of potential descriptors of water-soluble fullerene derivatives responsible for antitumor effects on lung cancer cells via QSAR analysis. *Computational and structural biotechnology journal* 19**,** 812-825.

Huang, Q., Wang, Y., Chen, X., Wang, Y., Li, Z., Du, S., Wang, L., and Chen, S. (2018). Nanotechnology-Based Strategies for Early Cancer Diagnosis Using Circulating Tumor Cells as a Liquid Biopsy. *Nanotheranostics* 2**,** 21-41.

Jayanthi, V.S.P.K.Sankara a., Das, A.B., and Saxena, U. (2017). Recent advances in biosensor development for the detection of cancer biomarkers. *Biosensors and Bioelectronics* 91**,** 15-23.

Li, S., Zhang, W., Xue, H., Xing, R., and Yan, X. (2020). Tumor microenvironment-oriented adaptive nanodrugs based on peptide self-assembly. *Chemical Science* 11**,** 8644-8656.

Liu, J., Li, Y., Yang, D., Yang, C., and Mao, L. (2019). Current state of biomarkers for the diagnosis and assessment of treatment efficacy of prostate cancer. *Discov Med* 27**,** 235-243.

Liu, Y., Zhu, F., Dan, W., Fu, Y., and Liu, S. (2014). Construction of carbon nanotube based nanoarchitectures for selective impedimetric detection of cancer cells in whole blood. *Analyst* 139**,** 5086-5092.

Maiti, D., Tong, X., Mou, X., and Yang, K. (2019). Carbon-Based Nanomaterials for Biomedical Applications: A Recent Study. *Frontiers in Pharmacology* 9.

Nicolini, A., Ferrari, P., and Duffy, M.J. (2018). Prognostic and predictive biomarkers in breast cancer: Past, present and future. *Seminars in Cancer Biology* 52**,** 56-73.

Ponomarenko, L.A., Schedin, F., Katsnelson, M.I., Yang, R., Hill, E.W., Novoselov, K.S., and Geim, A.K. (2008). Chaotic Dirac Billiard in Graphene Quantum Dots. *Science* 320**,** 356-358.

Qin, X., Liu, J., Zhang, Q., Chen, W., Zhong, X., and He, J. (2021). Synthesis of Yellow-Fluorescent Carbon Nano-dots by Microplasma for Imaging and Photocatalytic Inactivation of Cancer Cells. *Nanoscale Research Letters* 16**,** 14.

Sahoo, N.G., Bao, H., Pan, Y., Pal, M., Kakran, M., Cheng, H.K., Li, L., and Tan, L.P. (2011). Functionalized carbon nanomaterials as nanocarriers for loading and delivery of a poorly water-soluble anticancer drug: a comparative study. *Chem Commun (Camb)* 47**,** 5235-5237.

Santoni, G., Morelli, M.B., Amantini, C., and Battelli, N. (2018). Urinary Markers in Bladder Cancer: An Update. *Frontiers in oncology* 8**,** 362-362.

Sun, Q., Zhou, Z., Qiu, N., and Shen, Y. (2017). Rational Design of Cancer Nanomedicine: Nanoproperty Integration and Synchronization. *Advanced Materials* 29**,** 1606628.

Sun, Y., Zheng, S., Liu, L., Kong, Y., Zhang, A., Xu, K., and Han, C. (2020). The Cost-Effective Preparation of Green Fluorescent Carbon Dots for Bioimaging and Enhanced Intracellular Drug Delivery. *Nanoscale Research Letters* 15**,** 55.

Thakor, A.S., and Gambhir, S.S. (2013). Nanooncology: the future of cancer diagnosis and therapy. *CA Cancer J Clin* 63**,** 395-418.

Tian, P., Tang, L., Teng, K.S., and Lau, S.P. (2018). Graphene quantum dots from chemistry to applications. *Materials Today Chemistry* 10**,** 221-258.

Xi, G., Robinson, E., Mania-Farnell, B., Vanin, E.F., Shim, K.-W., Takao, T., Allender, E.V., Mayanil, C.S., Soares, M.B., Ho, D., and Tomita, T. (2014). Convection-enhanced delivery of nanodiamond drug delivery platforms for intracranial tumor treatment. *Nanomedicine: Nanotechnology, Biology and Medicine* 10**,** 381-391.

Zhang, C., Chen, X., Tan, L., and Wang, J. (2018a). Combined toxicities of copper nanoparticles with carbon nanotubes on marine microalgae Skeletonema costatum. *Environmental Science and Pollution Research* 25**,** 13127-13133.

Zhang, J., Zhao, X., Xian, M., Dong, C., and Shuang, S. (2018b). Folic acid-conjugated green luminescent carbon dots as a nanoprobe for identifying folate receptor-positive cancer cells. *Talanta* 183**,** 39-47.

Zhang, Y., Li, M., Gao, X., Chen, Y., and Liu, T. (2019). Nanotechnology in cancer diagnosis: progress, challenges and opportunities. *Journal of Hematology & Oncology* 12**,** 137.

Zhao, J., Luo, W., Xu, Y., Ling, J., and Deng, L. (2021). Potential reproductive toxicity of multi-walled carbon nanotubes and their chronic exposure effects on the growth and development of Xenopus tropicalis. *Science of The Total Environment* 766**,** 142652.
